# Unveiling the antitumor potential of novel N-(substituted-phenyl)-8-methoxycoumarin-3-carboxamides as dual inhibitors of VEGFR2 kinase and cytochrome P450 for targeted treatment of hepatocellular carcinoma

**DOI:** 10.3389/fchem.2023.1231030

**Published:** 2023-08-04

**Authors:** Eman M. Radwan, Eman Abo-Elabass, Atef E. Abd El-Baky, Hussah Abdullah Alshwyeh, Riyad A. Almaimani, Ghassan Almaimani, Ibrahim Abdel Aziz Ibrahim, Abdulaziz Albogami, Mariusz Jaremko, Samar Z. Alshawwa, Essa M. Saied

**Affiliations:** ^1^ The Division of Organic Chemistry, Chemistry Department, Faculty of Science, Port-Said University, Port-Said, Egypt; ^2^ The Division of Biochemistry, Chemistry Department, Faculty of Science, Port-Said University, Port-Said, Egypt; ^3^ Biochemistry Department, Faculty of Pharmacy, Port-Said University, Port-Said, Egypt; ^4^ Department of Biology, College of Science, Imam Abdulrahman Bin Faisal University, Dammam, Saudi Arabia; ^5^ Basic and Applied Scientific Research Centre, Imam Abdulrahman Bin Faisal University, Dammam, Saudi Arabia; ^6^ Department of Biochemistry, Faculty of Medicine, Umm Al-Qura University, Makkah, Saudi Arabia; ^7^ Department of Surgery, Faculty of Medicine, Umm Al-Qura University, Makkah, Saudi Arabia; ^8^ Department of Pharmacology and Toxicology, Faculty of Medicine, Umm Al-Qura University, Makkah, Saudi Arabia; ^9^ Biology Department, Faculty of science, Al-Baha University, Al Aqiq, Saudi Arabia; ^10^ Division of Biological and Environmental Sciences (BESE) and Engineering, King Abdullah University of Science and Technology (KAUST), Thuwal, Saudi Arabia; ^11^ Department of Pharmaceutical Sciences, College of Pharmacy, Princess Nourah Bint Abdulrahman University, Riyadh, Saudi Arabia; ^12^ Chemistry Department, Faculty of Science, Suez Canal University, Ismailia, Egypt; ^13^ Institute for Chemistry, Humboldt Universität zu Berlin, Berlin, Germany

**Keywords:** coumarin, hepatocellular carcinoma, cytotoxicity, cell arrest, apoptosis, cytochrome P450, VEGFR2

## Abstract

Being the sixth most diagnosed cancer and the fourth leading cause of cancer-related deaths worldwide, liver cancer is considered as a serious disease with a high prevalence and poor prognosis. Current anticancer drugs for liver cancer have drawbacks, such as limited efficacy in later stages of the disease, toxicity to healthy cells, and the potential for drug resistance. There is ample evidence that coumarin-based compounds are potent anticancer agents, with numerous analogues currently being investigated in preclinical and clinical studies. The current study aimed to explore the antitumor potency of a new class of 8-methoxycoumarin-3-carboxamides against liver cancer. Toward this aim, we have designed, synthesized, and characterized a new set of *N*-(substituted-phenyl)-8-methoxycoumarin-3-carboxamide analogues. The assessment of antitumor activity revealed that the synthesized class of compounds possesses substantial cytotoxicity toward Hep-G2 cells when compared to staurosporine, without significant impact on normal cells. Out of the synthesized compounds, compound **7** demonstrated the most potent cytotoxic effect against Hep-G2 cells with an IC_50_ of 0.75 µM, which was more potent than the drug staurosporine (IC_50_ = 8.37 µM). The investigation into the mechanism behind the antiproliferative activity of compound **7** revealed that it interferes with DNA replication and induces DNA damage, leading to cell cycle arrest as demonstrated by a significant decrease in the percentage of cells in the G1 and G2/M phases, along with an increase in the percentage of cells in the S phase. Flow cytometric analysis further revealed that compound **7** has the ability to trigger programmed cell death by inducing necrosis and apoptosis in HepG-2 cells. Further explorations into the mechanism of action demonstrated that compound **7** displays a potent dual-inhibitory activity toward cytochrome P450 and vascular endothelial growth factor receptor-2 (VEGFR-2) proteins, as compared to sorafenib drug. Further, detailed computational studies revealed that compound **7** displays a considerable binding affinity toward the binding cavity of VEGFR2 and CYP450 proteins. Taken together, our findings indicate that the newly synthesized class of compounds, particularly compound **7**, could serve as a promising scaffold for the development of highly effective anticancer agents against liver cancer.

## 1 Introduction

Cancer continues to be a significant global health challenge, with millions of people affected by various types of cancer each year (Kocarnik et al., 2022; Tran et al., 2022). Among the many types of cancer, liver cancer stands out as a particularly concerning condition due to its high prevalence and poor prognosis. Liver cancer, also known as hepatocellular carcinoma (HCC), is a type of cancer that starts in the cells of the liver and can spread to other parts of the body. Liver cancer is a major public health issue worldwide. According to the World Health Organization (WHO), liver cancer is the sixth most commonly diagnosed cancer and the fourth leading cause of cancer-related deaths globally (Ferlay et al., 2019; Rumgay et al., 2022). Treatment options for liver cancer depend on the severity and stage of the disease. They may include surgery (such as liver resection or transplantation), radiation therapy, chemotherapy, targeted therapies, and immunotherapies. However, the effectiveness of treatment depends on the stage of liver cancer at the time of diagnosis, and the availability of resources and expertise for managing this complex condition (Liu et al., 2015; Medavaram and Zhang, 2018; Llovet et al., 2021).

VEGFR-2, also known as vascular endothelial growth factor receptor-2, is a protein that belongs to the family of receptor tyrosine kinases (RTKs) and involves in the progression and development of liver cancer, as angiogenesis is a crucial process in metastasis and tumor growth (Shibuya, 2011; Modi and Kulkarni, 2019). As a member of the VEGF receptor family, VEGFR-2 is a transmembrane receptor that is primarily expressed on the surface of endothelial cells, which are the cells that line blood vessels. In liver cancer, VEGFR-2 has been found to be overexpressed, meaning that there is an increased amount of this receptor present compared to normal liver tissue (Shibuya, 2011; Apte et al., 2019). Binding of the key angiogenic factor, vascular endothelial growth factor (VEGF), to VEGFR-2 triggers a cascade of intracellular events that promote capillary tube formation, migration, and endothelial cell proliferation. This leads to the formation of new blood vessels, which provide oxygen and nutrients to the growing tumor mass (Hicklin and Ellis, 2005; Schoenleber et al., 2009; Chen et al., 2016). In addition to angiogenesis, VEGFR-2 signaling can promote tumor cell survival, invasion, and metastasis, as well as modulate the tumor microenvironment by affecting immune cell recruitment and function. Targeting VEGFR-2 has emerged as a possible therapeutic strategy for liver cancer. Several anti-angiogenic drugs that specifically inhibit VEGFR-2 activity, such as regorafenib, lenvatinib, and sorafenib have been clinically approved for the advanced HCC treatment (Zhu et al., 2020; Niu et al., 2021). These drugs can block the binding of VEGF to VEGFR-2, thereby inhibiting angiogenesis and tumor vascularization, and potentially reducing tumor growth and metastasis (Bruix et al., 2017). While drugs that target VEGFR-2 have shown promise as a therapeutic approach for liver cancer, they also have some potential disadvantages, including, limited efficacy, adverse effects, off-target effects, development of resistance, cost and accessibility, and lack of long-term data. Therefore, finding potential antitumor agents against liver cancer that could target the intricate mechanisms of VEGFR-2 signaling with minor adverse effects is urgently needed (Huang et al., 2020; Luo et al., 2021).

Cytochrome P450 2D6 (CYP2D6) is an enzyme that plays a crucial role in drug metabolism and is primarily expressed in the liver. It is responsible for the metabolism of a wide range of drugs, including many commonly prescribed medications and other xenobiotics (Zanger and Schwab, 2013; Zhao et al., 2021). Multiple lines of evidence suggest that CYP2D6 may be involved in various mechanisms that could potentially contribute to the progression of liver cancer (Hu G. et al., 2021; Khamis et al., 2021). CYP2D6 is known to metabolize several drugs used in the treatment of liver cancer, such as tamoxifen, codeine, and oxycodone. Altered CYP2D6 activity due to genetic variations or other factors could impact the metabolism of these drugs, potentially affecting their efficacy or toxicity (Taylor et al., 2020; Dean and Kane, 2021). Further, CYP2D6 may interact with other enzymes involved in drug metabolism, such as CYP3A4 and CYP2C9, which are also expressed in the liver (Tarantino et al., 2009; Hakkola et al., 2020). Based on these facts, targeting CYP2D6 has emerged as a potential therapeutical approach for liver cancer.

**FIGURE 1:**
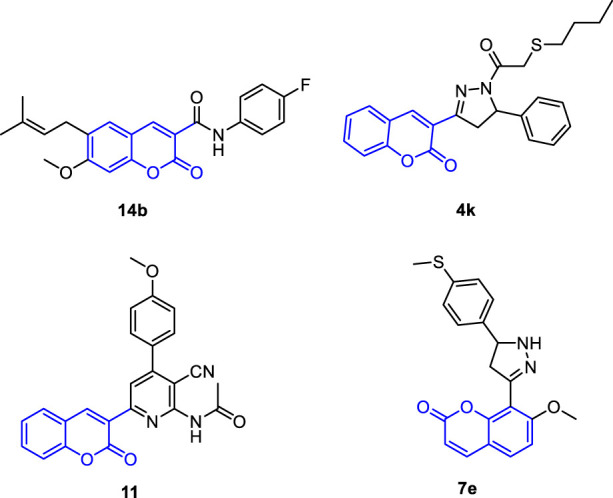
Representative reported coumarin analogues with potent antitumor activity against liver cancer cell line.

The discovery and development of potent drugs with minimal harmful side effects is a primary goal of modern medicinal chemistry. Coumarins are a class of bioactive molecules that are also known as *cis*-*O*-hydroxycinnamic lactones and possess a benzo-α-pyranone moiety in their basic structure (Küpeli Akkol et al., 2020; Salehian et al., 2021). They are secondary metabolites found in plants, bacteria, and fungi, with approximately 1,300 types of coumarins recognized so far (Stefanachi et al., 2018; Viana et al., 2021). Coumarin-based compounds have gained increasing attention due to their broad range of biological activities (Ahmed et al., 2020; Küpeli Akkol et al., 2020; Wu et al., 2020; Rawat and Vijaya Bhaskar Reddy, 2022). They have been reported to exhibit anticoagulant (Lu et al., 2022), antibacterial (Liu H. et al., 2020; Qin et al., 2020; Hu Y. et al., 2021), anti-inflammatory (Liang et al., 2020; Nayeli et al., 2020; Wang et al., 2020; Alfayomy et al., 2021), antioxidant (Sanches et al., 2019; Li et al., 2020; Ozalp et al., 2020; Parvin et al., 2021), antitumor (Kaur et al., 2015; Mohammed et al., 2020; Shahzadi et al., 2020; Konkoľová et al., 2021; Zhang et al., 2021; Rawat and Vijaya Bhaskar Reddy, 2022), antiviral (Liu G.-L. et al., 2020; Chidambaram et al., 2021; Shan et al., 2021; Özdemir et al., 2022), hyperlipidemia (Miao et al., 2021), anti-Alzheimer (Francisco et al., 2020), and enzyme inhibition effects (Zengin Kurt et al., 2019; Supuran, 2020; Xu et al., 2020; Meleddu et al., 2021). In the context of cancer treatment, coumarin analogues have been gaining increasing attention in recent years due to their potential as anticancer agents against liver cancer (Küpeli Akkol et al., 2020; Wu et al., 2020; Rawat and Vijaya Bhaskar Reddy, 2022). Coumarin derivatives (pyrazole, furan, sulfonyl, azoles, etc) have been found to promote cell cycle arrest, kinase inhibition, carbonic anhydrase inhibition, angiogenesis inhibition, and telomerase inhibition in different types of cancer cells. The specific substitution pattern on the coumarin ring governs its therapeutic applications and pharmacological properties (Küpeli Akkol et al., 2020; Wu et al., 2020; Rawat and Vijaya Bhaskar Reddy, 2022). As such, there is growing interest in the applications and future prospects of coumarin analogues as anticancer agents against liver cancer. For example, Phutdhawong *et al.* synthesized novel coumarin-3-carboxamides and found that compound **14b** possess potent anti-cancer potential against HepG-2 and HeLa cancer cell lines (Phutdhawong et al., 2021). Additionally, new coumarin derivatives have been synthesized and screened for anticancer potential against different cancer cell lines. Among synthesized compounds, compound **11** demonstrated a potential cytotoxic activity against HepG-2 cells with IC_50_ of 4.5 uM (Figure 1) (Fayed et al., 2019). A study conducted by Wu et al. investigated the effect of the introduction of the dihydropyrazole moiety in the coumarin skeleton. The authors showed that this class of coumarin analouges possesses considerable anticancer properties of coumarin analogs by inducing apoptosis and targeting telomerase activity e.g., compound **4k**). The findings of this study suggest that coumarin analogues may have potential therapeutic applications in the treatment of liver diseases. In a study by Fayed et al. (2019), it was shown that coumarin analogues with pyridine hybrids possess a wide range of anti-cancer properties. These compounds demonstrated the capacity to induce apoptosis and cell cycle arrest, along with a significant enhancement in caspase-3 activity (e.g., compound **11**).

**FIGURE 2:**
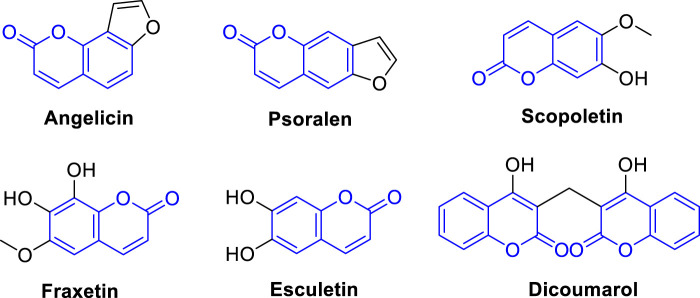
Representative coumarin analogues under preclinical or clinical investigations with anti-cancer activity against HCC.

Currently, there are several coumarin analogues that have been studied for their anti-liver cancer activity in preclinical or clinical, including furanocoumarin, umbelliprenin, scopoletin, fraxetin, esculetin, and dicoumarol (Figure 2) (Annunziata et al., 2020; Küpeli Akkol et al., 2020; Wu et al., 2020; Rawat and Vijaya Bhaskar Reddy, 2022). Furanocoumarins are a group of coumarin analogues found in certain plants, such as citrus fruits and herbs like *Angelica archangelica* (Bruni et al., 2019). Some furanocoumarins, such as psoralen and angelicin, have shown anti-liver cancer activity in preclinical studies by inducing cell cycle arrest and apoptosis in liver cancer cells (Ahmed et al., 2020). Scopoletin, also known as coumarin-7-hydroxy, is a coumarin analogue found in several medicinal plants (Antika et al., 2022). Some studies have shown that scopoletin exhibits anti-liver cancer activity by inhibiting cell proliferation, inducing apoptosis, and inhibiting angiogenesis in liver cancer cells (Wang et al., 2012; Sabeel et al., 2023). Dicoumarol is a naturally occurring coumarin analogue found in various plant species (Silva et al., 2022). It has been reported to possess anti-liver cancer activity by inhibiting the NAD(P)H:quinone oxidoreductase 1 (NQO1) enzyme, which is involved in tumor growth and survival pathways (Cullen et al., 2003). Esculetin is a coumarin analogue that has been shown to have anti-liver cancer properties. It has been reported to inhibit cell proliferation, induce apoptosis, and inhibit metastasis of liver cancer cells (Wang et al., 2015). Esculetin has also been shown to have anti-angiogenic effects in liver cancer, which can inhibit tumor growth and metastasis (Arora et al., 2016; Garg et al., 2022). Fraxetin is a coumarin analogue that has been studied for its potential anti-liver cancer activities. It has been reported to inhibit cell proliferation, induce apoptosis, and inhibit invasion and migration of liver cancer cells. Fraxetin has also been shown to modulate various signaling pathways involved in liver cancer development and progression (Figure 2) (Song et al., 2021). Accordingly, the diverse applications of coumarin analogues make them a promising avenue in future research for drug discovery and development (Annunziata et al., 2020; Küpeli Akkol et al., 2020; Wu et al., 2020; Rawat and Vijaya Bhaskar Reddy, 2022). In addition, the ease of synthesis and modifiability of the coumarin scaffold makes it a versatile platform for the design of novel compounds with improved pharmacological properties (Sarmah et al., 2021; Chaudhary et al., 2022; Sharma et al., 2022).

**FIGURE 3:**
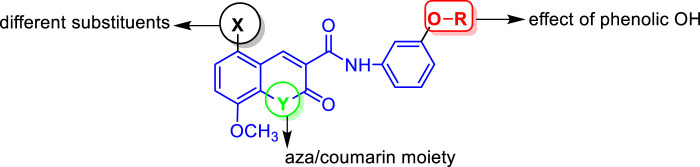
Representative structural features examined in the designed *N*-(substituted-phenyl)-8-methoxycoumarin-3-carboxamide analogues.

Based on the abovementioned facts and our continuous interest in discovering novel bioactive probes (Banhart et al., 2014; Saied et al., 2014; Saied et al., 2015; Saied et al., 2018; Gaber et al., 2020; El Azab et al., 2021; Saied and Arenz, 2021; Khirallah et al., 2022a; Khirallah et al., 2022b; Healey et al., 2022; Salem et al., 2022), the objective of this study was to design and synthesize a new series of *N*-(substituted-phenyl)-8-methoxycoumarin-3-carboxamides, and evaluate their cytotoxic activity against liver cancer cells (Figure 3). Additionally, the study aimed to explore the mechanism underlying the cytotoxic effects by assessing programmed cell death, cell cycle arrest, as well as inhibitory activity against CYP2D6 and VEGFR-2. Furthermore, *in silico* molecular modeling was performed to explore the binding potency of these compounds to the active site of VEGFR-2 and CYP2D6 proteins.

## 2 Results and discussion

### 2.1 Synthesis and characterization of 8-methoxycoumarin-3-carboxamides

**SCHEME 1:**
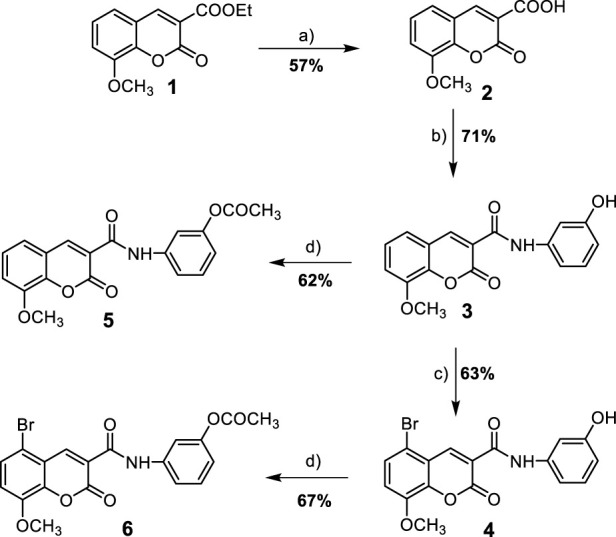
Synthesis of 8-methoxycoumarin-3-carboxamide derivatives **(3–6)**. Reagent and conditions: (a) 4N HCl, AcOH, reflux-rt., 18 h; (b) i- SOCl_2_, reflux, 2 h, ii- 3-aminophenol, DMF, reflux-r.t., 12 h; (c) Br_2_, AcOH, 60°C-r.t., 18 h; (d) Ac_2_O, reflux-r.t., 16 h.

In the present work, a series of *N*-(substituted)phenyl-8-methoxycoumarin-3-carboxamides, azacoumarin-3-carboxamide and their brominated derivatives (**3–8**) were synthesized from ethyl 8-methoxycoumarin-3-carboxylate (**1**) through a multi-step reaction sequence as shown in Scheme 1, 2. The synthesis of the target compounds involved several key steps, including cyclocondensation, hydrolysis, acid chloride formation, condensation, bromination, and acetylation, which were carefully designed and executed to obtain the desired products. The starting material, ethyl 8-methoxycoumarin-3-carboxylate (**1**), was synthesized in a satisfactory yield (86%) from 3-methoxy-2-hydroxybenzaldehyde and diethyl malonate through Knovanaegel condensation in the presence of piperidine as a base catalyst (Wu et al., 2014). The formation of compound **1** was affirmed by spectroscopic techniques such as ^1^H nuclear magnetic resonance (NMR), ^13^C NMR, infrared (IR), and mass spectrometry (EI-MS). Compound **1** was then converted to 8-methoxycoumarin-3-carboxylic acid (**2**) through hydrolysis with 4N HCl in acetic acid under reflux conditions to furnish compound **2** in 57% yield. The hydrolysis reaction was monitored by TLC and the formation of compound **2** was confirmed by spectroscopic techniques. Compound **2** was then used as a key intermediate for the synthesis of various derivatives. *N*-(3-hydroxy)phenyl 8-methoxycoumarin-3-carboxamide (**3**) was synthesized from compound **2** through the reaction with thionyl chloride to yield coumarin-3-acid chloride, followed by condensation with 3-aminophenol to afford compound **3** in a good yield (Healey et al., 2022). To obtain the brominated derivative, *N*-(3-hydroxy)phenyl 5-bromo-8-methoxycoumarin-3-carboxamide (**4**), compound **3** was brominated with bromine in glacial acetic acid with stirring at 60°C to provide compound **4** in a moderate yield (63%) (Khirallah et al., 2022a). The bromination reaction was monitored by TLC and the formation of compound 4 was confirmed by spectroscopic techniques. To further support the structures of compounds **3** and **4**, they were transformed into *N*-(3-acetoxy)phenyl 8-methoxycoumarin-3-carboxamide (**6**) through acetylation with boiling acetic anhydride to give compound **6** in 67% yield (Khirallah et al., 2022b). The formation of compound **6** was also confirmed by spectroscopic techniques.

**SCHEME 2:**
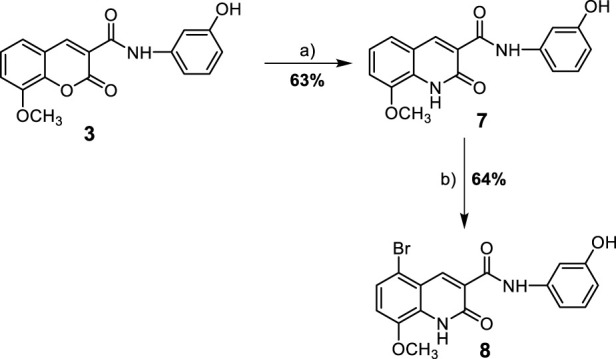
Synthesis of 8-methoxy-azacoumarin-3-carboxamide derivatives **(7 and 8)**. Reagent and conditions: (a) anhyd. K_2_CO_3_, NH_3_ (soln.), reflux, 6 h; (b) Br_2_, AcOH, 60°C-r.t., 18 h.

In addition to the coumarin derivatives, the synthesis of aza-coumarin analogues was also attempted (Scheme 2). *N*-(3-hydroxy)phenyl 8-methoxycoumarin-3-carboxamide (**3**) was converted to *N*-(3-hydroxy)phenyl-8-methoxy-azacoumarin-3-carboxamide (**7**) through aminolysis with ammonia solution in ethanol in the presence of anhydrous potassium carbonate under reflux to afford compound **7** in a good yield (63%). This was followed by halogenation of compound **7** with bromine in glacial acetic acid, resulting in the formation of *N*-(3-hydroxy)phenyl 5-bromo-8-methoxy-azacoumarin-3-carboxamide (**8**) in 64% yield, as shown in Scheme 2.

The successful synthesis of the targeted coumarin and aza-coumarin derivatives demonstrates the efficacy of the multi-step reaction sequence employed in this study (Bakare, 2021; Chaudhary et al., 2022; Szwaczko, 2022). The halogenation reactions leading to the formation of compounds **4** and **8** highlight the versatility of the synthetic route in introducing bromine substitution at the desired positions. The attempted synthesis of aza-coumarin analogues (compounds **7** and **8**) represents a novel approach in this study. The conversion of the coumarin scaffold to the corresponding aza-coumarin scaffold was successfully achieved through treatment with ammonia solution, followed by halogenation.

### 2.2 Characterization of synthesized compounds

The structures of compounds **3-6** were confirmed by various spectroscopic techniques including IR, ^1^H-NMR, ^13^C-NMR, and mass spectrometry (EI-MS) which provided evidence for their chemical identity and purity. In the IR spectra, characteristic absorption bands corresponding to specific functional groups such as carbonyl, amide, and bromine were observed, which supported the presence of these groups in the synthesized compounds. The ^1^H-NMR and ^13^C-NMR spectra provided detailed information about the chemical shifts of different protons and carbons in the molecules, which further confirmed the structures of the compounds. The mass spectrometry (EI-MS) data showed the expected molecular ion peaks and fragmentation patterns, which were consistent with the proposed structures. The ^1^H-NMR spectrum of compound **3** exhibited several signals that provided information about its chemical structure. There were two singlet signals observed at δ 10.59 and 9.60 ppm, which can be attributed to the protons of the NH and OH groups, respectively. Another singlet signal at δ 8.87 ppm was observed, which can be assigned to the proton of H-4 in the coumarin ring. Furthermore, the absence of proton signals at δ 1.33 ppm suggests the absence of the acetoxy group (OCH_2_CH_3_) in compound **3**. This is supported by the appearance of a singlet signal at δ 3.96 ppm, which can be attributed to the protons of the methoxy group (OCH_3_). The proton signals of the aromatic rings in compound **3** were observed within the expected chemical shifts in the region of δ 6.57–7.54 as multiplet signals. This confirms the presence of aromatic rings in the compound. The ^13^C-NMR spectrum of compound **3** revealed several carbon signals that provide further information about its chemical structure. There were two carbon signals observed at δ 160.72 and 160.14 ppm, which can be attributed to the carbonyl groups of the amide and pyranone ring, respectively. Another carbon signal at δ 56.70 ppm was observed, which can be assigned to the carbon of the methoxy group (OCH_3_). This is consistent with the presence of a methoxy group in compound **3**. The carbon signals detected within δ 158.28–107.32 ppm further confirm the presence of aromatic and coumarin rings in compound **3**. Overall, the ^1^H NMR and ^13^C NMR spectra of compound **3** provide clear evidence for its chemical structure. The detected signals corresponding to the OH and NH groups, H-4 of the coumarin ring, methoxy group, and aromatic rings are consistent with the expected chemical shifts, confirming the presence of these functional groups and rings in compound **3**.

The ^1^H NMR spectra of compounds **5** and **6** provide important information about their chemical structures. The disappearance of a proton signal at δ 9.60 ppm suggests the absence of a hydroxyl group (OH) in both compounds. Instead, new singlet signals were observed at δ 2.30 and 2.38 ppm, which can be attributed to the three protons of the methyl group in the acetoxy group (OCOCH_3_). This indicates that compounds **5** and **6** have undergone acetylation with acetic anhydride, resulting in the formation of acetoxy groups. Additionally, the NH and H-4 proton signals of the coumarin ring were still present in compounds **5** and **6**, observed at δ 10.77, 11.30 and 8.88, 9.01 ppm, respectively. This confirms that the coumarin ring is still present in these compounds. The methoxy (OCH_3_) groups appeared as singlet signals at δ 3.98–3.96 ppm, indicating the presence of methoxy groups in compounds **5** and **6**. The aromatic protons were detected as multiplet signals within the expected chemical shifts, further supporting the presence of aromatic rings in compounds **5** and **6**. The ^13^C NMR spectra of compounds **5** and **6** revealed the presence of two new carbon signals at δ 169.67, 168.64 ppm and 21.35, 20.98 ppm, which can be attributed to the carbonyl carbons of the acetoxy groups (OCOCH_3_). This provides evidence for the acetylation of compounds **3** and **4**, resulting in the formation of compounds **5** and **6**. The ^1^H and ^13^C NMR spectra of compounds **5** and **6** provide important information about their chemical structures. The absence of a proton signal for the hydroxyl group and the appearance of singlet signals for the methyl group in the acetoxy group confirm the acetylation of compounds **3** and **4**. The presence of NH and H-4 proton signals of the coumarin ring, methoxy groups, and aromatic protons further support the chemical structures of compounds **5** and **6.** The ^13^C NMR spectra provide additional evidence for the presence of acetoxy groups in compounds **5** and **6**.

The mass spectra of compounds **3** and **5** show prominent molecular ion peaks at *m/z* 311 and m/z 353, respectively. These peaks correspond to the molecular formula C_17_H_13_NO_5_ for compound **3** and C_19_H_15_NO_6_ for compound **5**. The molecular ion peak represents the intact molecular ion of the compound, and its *m/z* value provides information about the mass of the compound and its composition. The presence of molecular ion peaks at *m/z* 311 and *m/z* 353 in the mass spectra of compounds **3** and **5**, respectively, suggests that these compounds have relatively stable molecular ions. This indicates that the molecular ions of compounds **3** and **5** are less likely to undergo fragmentation or other chemical reactions during the ionization process. On the other hand, it was observed that the molecular ion peaks of compounds **4** and **6** are unstable. This means that the molecular ions of compounds **4** and **6** are not as stable as those of compounds **3** and **5**, and they may be prone to fragmentation or other chemical reactions during the ionization process (Supplementary Figures S1, S2). This instability could be due to the presence of labile functional groups or other structural features in compounds **4** and **6** that make their molecular ions less stable.

### 2.3 Evaluation of cytotoxic activity against liver cancer cells

**FIGURE 4:**
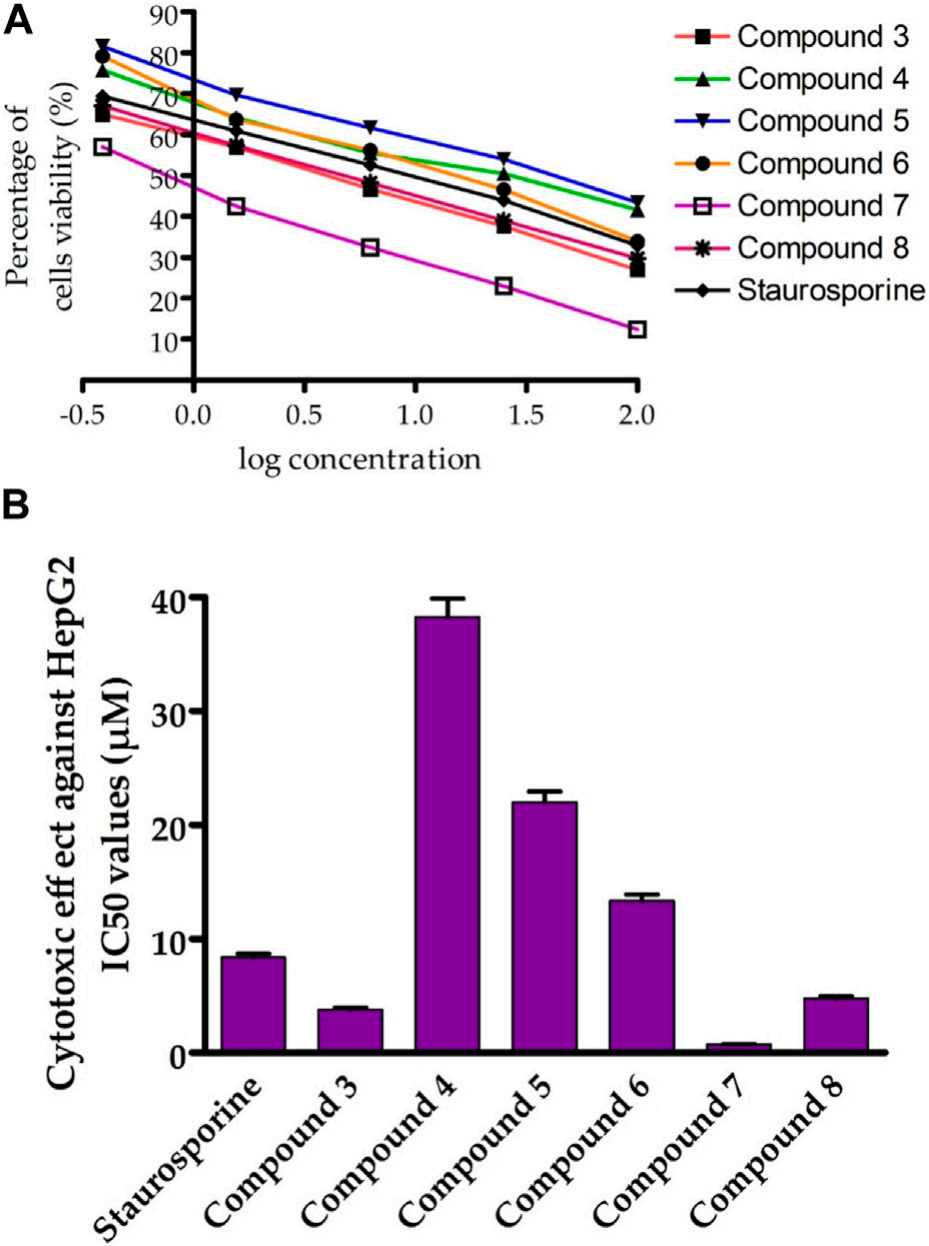
Assessment of cytotoxic activity of synthesized compounds toward Hep-G2 cells. **(A)** the dose-dependent cytotoxic activity of synthesized compounds. **(B)** Representative diagram for the IC_50_ (µM) values of synthesized compounds against Hep-G2 cells proliferation, as compared to STU. The data presented represents the mean ± SEM derived from the dose-response curve of a minimum of three independent experiments.

We first screened the cytotoxicity of compounds against the proliferation of liver cancer cells (HepG-2) by using the MTT assay (Mohamed et al., 2022a). Toward this, the HepG-2 cells were cultured and treated with tested compounds at different concentrations for 24 h and subsequently 3-(4,5-dimethylthiazol-2-yl)-2,5-diphenyltetrazolium bromide (MTT) dye was added. The colorimetric measurement was assessed using an ELISA plate reader at a wavelength of 570 nm. In this assay, the activity of compounds was compared to the standard anticancer staurosporine (STU) drug under identical experimental conditions. As indicated in Figure 4; Table 1, the synthesized compounds exhibited considerable anti-proliferative activity against HepG-2 cells. The N-(3-hydroxy)phenyl 8-methoxycoumarin-3-carboxamide **3** exhibited a significant cytotoxic activity with IC_50_ of 3.81 uM. The bromine substitution at the 8-methoxycoumarin moiety of compound **3** provided a compound with lower inhibitory activity toward the growth of HepG-2 cells (compound 4, IC_50_ 38.28 uM). This result indicates that the introduction of the large bromine atom may attenuate the binding affinity of the compound toward the targeted protein (s).

**TABLE 1:** Cytotoxic evaluation of compounds **3-8 against human liver carcinoma HepG2 cell line and HL-7702 normal cell line.**

Comp no.	IC_50_ values (µM)	
	HepG2	HL-7702
3	3.81 ± 0.16	NT^a^
4	38.28 ± 1.62	NT^a^
5	22.03 ± 0.93	NT^a^
6	13.37 ± 0.56	NT^a^
7	0.75 ± 0.03	13.72 ± 1.8
8	4.79 ± 0.2	NT^a^
STU	8.37 ± 0.35	22.17 ± 2.1

^a^ NT, not determined.

Further, acylation of the hydroxyl group at the phenyl 3-carboxamide moiety of compound **3** resulted in a considerable diminish in the cytotoxic activity (compound **5**, IC_50_ 22.03 µM), indicating the significance of the phenolic hydroxyl group in the antiproliferative activity of compound **3**. Conversely, acylation of 3-hydroxy-phenyl moiety in compound **5** led to a substantial improvement in the antiproliferative activity of the compound (compound **6**, IC_50_ 13.37 µM). Interestingly, shifting the 8-methoxycoumarin moiety in compound **3** into 8-methoxy-azacoumarin analogue (compound **7**) substantially improved the cytotoxic activity (IC_50_ 0.75 ± 0.03 µM), revealing the significance of the amine group in the cytotoxicity of this class of compounds. Similarly, bromination of compound **7** at the 8-methoxycoumarin-3-carboxamide moiety resulted in a dramatic diminish in the cytotoxic effect of the compound (IC_50_ 4.79 ± 0.2 µM). These results further demonstrate that the bromination of 8-methoxycoumarin-3-carboxamide moiety has a drastic effect on the antiproliferative of this class of compounds. Subsequently, we investigated the cytotoxic effects of these compounds on human normal liver cells (HL-7702). As indicated in Table 1, the synthesized compounds, except for compound **7**, did not show substantial cytotoxic activity toward human normal liver cells, demonstrating that this class of compounds exhibits a substantial and selective antiproliferative activity against HepG-2 cells, with a non-dramatic cytotoxic effect on normal liver cells. These findings align with previous studies that have demonstrated that methoxy-coumarin analogues exhibit potential anti-proliferative activity (Amin et al., 2015; Küpeli Akkol et al., 2020; Wu et al., 2020; Rawat and Vijaya Bhaskar Reddy, 2022). Among synthesized and investigated compounds, compound **7** demonstrated the most antiproliferative activity HepG-2 cells with IC_50_ of 0.75 ± 0.03 µM, as compared to STU drug (IC_50_ 8.37 ± 0.35 µM). In addition, compound **7** did not show a considerable cytotoxic activity against HL-7702 cells (IC_50_ 13.72 µM), as compared to STU drug with IC_50_ of 22.17 µM. Overall, these findings indicate that this class of 8-methoxycoumarin-3-carboxamide compounds could be considered for the development of promising antiproliferative lead compounds against hepatocellular carcinoma.

### 2.4 Cell cycle analysis

**FIGURE 5:**
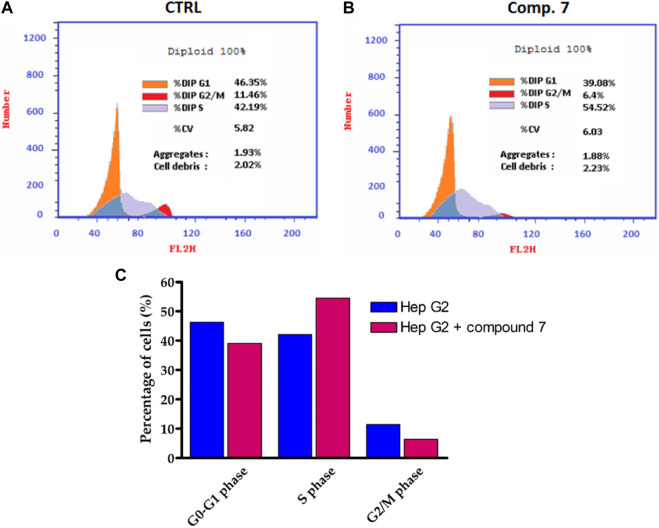
Influence of compound **7** at a concentration of 0.75 µM on Hep-G2 cell distribution. **(A,B)** Flow cytometry assessment of cell cycle stages in Hep-G2 cells of untreated and compound **7**-treated and cells. **(C)** Cell cycle distribution histograms of treated and untreated Hep-G2 cells.

Among the compounds tested, our findings revealed that compound **7** displayed the highest level of cytotoxic activity against the liver carcinoma cell line (HepG-2). Based on this promising activity, compound **7** was selected for further evaluation to assess its effect on the cell cycle profile in HepG-2 cells. To evaluate the cell cycle, a biparametric cytofluorimetric analysis was conducted on HepG-2 cells treated with compound **7** at its IC_50_ concentration for a duration of 24 h. Propidium iodide (PI) was used as the staining agent for this analysis. (Figure 5). The analysis revealed that Compound 7 caused a significant decrease in the percentage of cells in the G1 and G2/M phases of the cell cycle. In the untreated control group, 46.35% of cells were in the G1 phase, while after treatment with compound **7**, the percentage decreased to 39.08%. Similarly, the percentage of cells in the G2/M phase decreased from 11.46% in the control group to 6.4% after treatment with compound **7**. In contrast, there was a notable increase in the percentage of cells in the S phase after treatment with Compound 7. The S phase, which is responsible for DNA synthesis, showed an increase from 42.19% in the untreated control group to 54.52% after treatment with Compound **7**. These findings indicate that the treatment of HepG-2 cells with compound **7** leads to cell cycle arrest specifically in the S phase. This is evident from the decrease in the proportion of cells in the G1 and G2/M phases, accompanied by an increase in the percentage of cells residing in the S phase. Cell cycle arrest is a widely recognized mechanism utilized by anticancer agents to impede the excessive proliferation of cancer cells. By inducing cell cycle arrest, these agents disrupt the normal progression of the cell cycle, preventing cancer cells from dividing and multiplying uncontrollably. This therapeutic strategy helps to halt tumor growth and promotes the effectiveness of cancer treatments. The observed effects of compound **7** on the cell cycle profile of HepG-2 cells are indicative of its potential as an antitumor agent. The decrease in the percentage of cells in the G1 phase suggests that compound **7** may inhibit the progression of cells from the G1 to S phase, which could lead to cell cycle arrest and subsequent inhibition of cell proliferation. Additionally, the decrease in the percentage of cells in the G2/M phase suggests that Compound **7** may also affect cell division and mitotic progression. Furthermore, the increase in the percentage of cells in the S phase upon treatment with Compound **7** may indicate DNA damage and activation of DNA repair mechanisms, which can contribute to the observed S phase arrest. This suggests that Compound **7** may interfere with DNA replication and induce DNA damage, leading to cell cycle arrest and inhibition of tumor cell growth. Overall, these results highlight the potential of Compound **7** as a promising antitumor agent against liver carcinoma, as it exhibits potent cytotoxic activity and induces S phase arrest in HepG-2 cells.

### 2.5 Flow cytometric analysis

**FIGURE 6:**
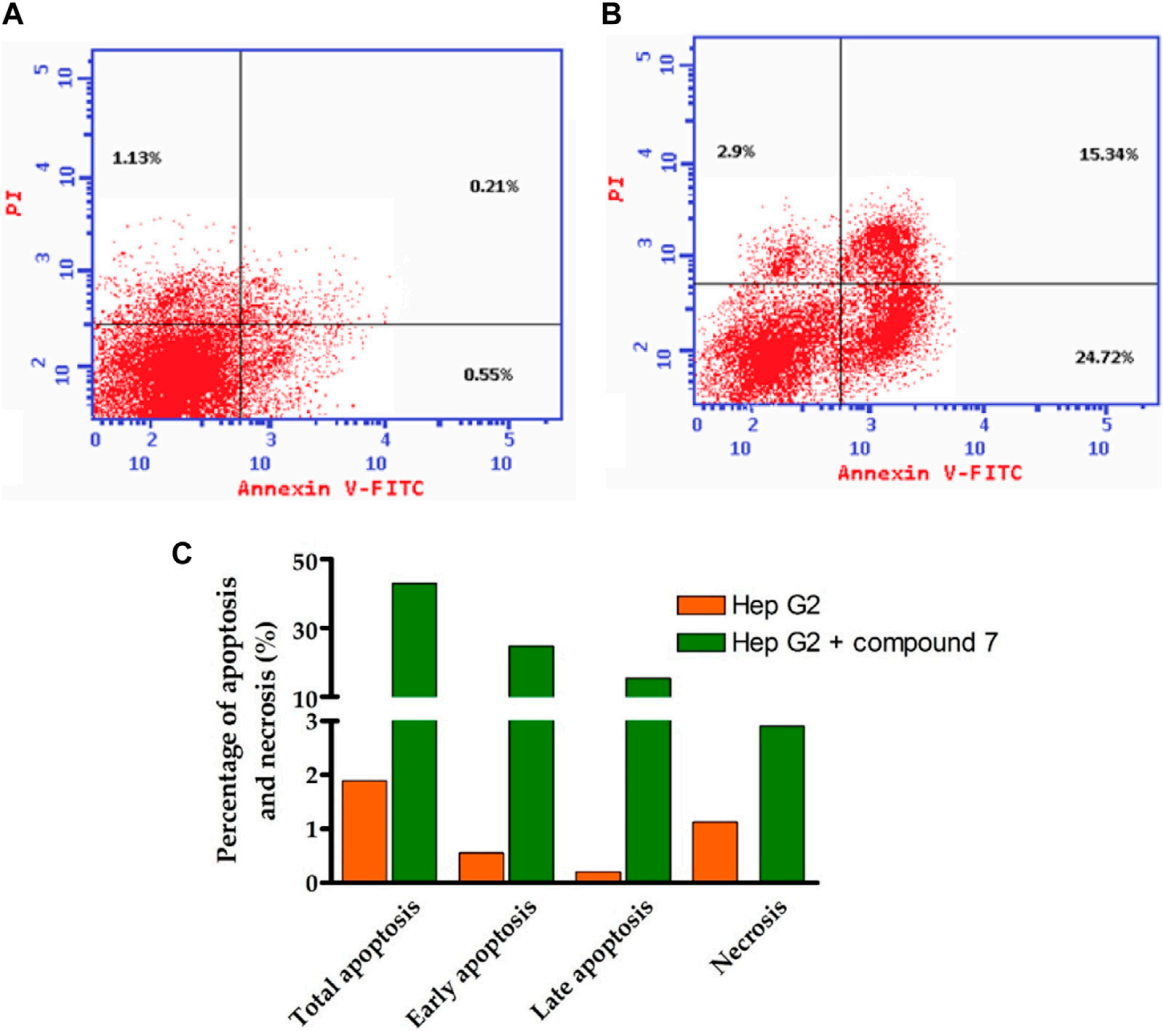
Illustrates the influence of compound 7 on programmed cell death in Hep-G2 cells. **(A)** The cytofluorometric analysis of untreated Hep-G2 cells was performed using the Annexin V FITC double labeling assay. **(B)** The Annexin V FITC double labeling assay was utilized to analyze Hep-G2 cells treated with compound 7 at a concentration of 0.75 µM. **(C)** A graphical representation is provided, demonstrating the different stages of programmed cell death in both untreated Hep-G2 cells and those treated with compound 7.

The Annexin V-FITC/PI assay is a well-established flow cytometric method to evaluate the apoptotic potential of bioactive compounds. The assay allows differentiation between apoptotic cells and live cells by using fluorescent dyes that bind to phosphatidylserine (PS) and DNA, respectively (Galluzzi et al., 2018; Pfeffer and Singh, 2018; Tong et al., 2022). In the present study, the assay was performed to explore the apoptotic effect of compound **7** (at its IC_50_ dose value) on HepG-2 cells. As indicated in Figure 6, the results revealed a substantial increase in the percentage of apoptotic cells at all stages (total, early, and late) compared to the control group. In this regard, compound **7** displayed the ability to increase the percentage of total apoptotic from (42.96%) by 23-fold compared to the control group (1.89%). At the early stage, the percentage of apoptotic cells increased from 0.55% in the control group to 24.72% in the compound **7**-treated group (45-fold increase). Similarly, at the late stage, the percentage of apoptotic cells increased from 0.21% in the control group to 15.34% in the compound **7**-treated group (73-fold increase). These results clearly demonstrate that compound **7** is able to induce apoptosis in these cells. Further, our results indicated that compound **7** exhibits a considerable ability to induce necrosis (2.5- fold increase) in HepG-2 cells, as compared to the control cells. The Annexin V-FITC/PI assay is a well-established method to evaluate the apoptotic potential of chemical compounds. Inducing apoptosis is an appealing therapeutic approach for cancer treatment as it is a vital cellular process essential for preserving tissue balance and removing impaired or unnecessary cells. Disruption in the regulation of apoptosis can contribute to various diseases, such as cancer, highlighting its significance. Hence, triggering apoptosis holds potential as an effective strategy to combat cancer (Galluzzi et al., 2018; Pfeffer and Singh, 2018; Tong et al., 2022). The presented results suggest that compound **7** may have potential as an anti-cancer agent by inducing apoptosis and necrosis cell death in HepG-2 cells. The observed increase in the percentage of apoptotic cells at all stages (total, early, and late) compared to the control group indicates that compound **7** is able to activate the apoptotic pathway in these cells. The early stage of apoptosis is characterized by the translocation of PS from the inner to the outer leaflet of the plasma membrane, which can be detected by Annexin V-FITC staining. In the present study, the percentage of Annexin V-FITC-positive cells significantly increased in the compound **7**-treated group, indicating that compound **7** induces early apoptosis in HepG-2 cells. Further, the late stage of apoptosis is characterized by DNA fragmentation, which can be detected by PI staining. Our findings revealed that the percentage of PI-positive cells significantly increased in the compound **7**-treated group, indicating that compound **7** induces late apoptosis in HepG-2 cells. According to these findings, there is a notable connection between the ability of compound **7** to induce programmed cell death in HepG-2 cells and its antiproliferative effects.

### 2.6 Assessment of VEGFR-2 inhibitory activity

**FIGURE 7:**
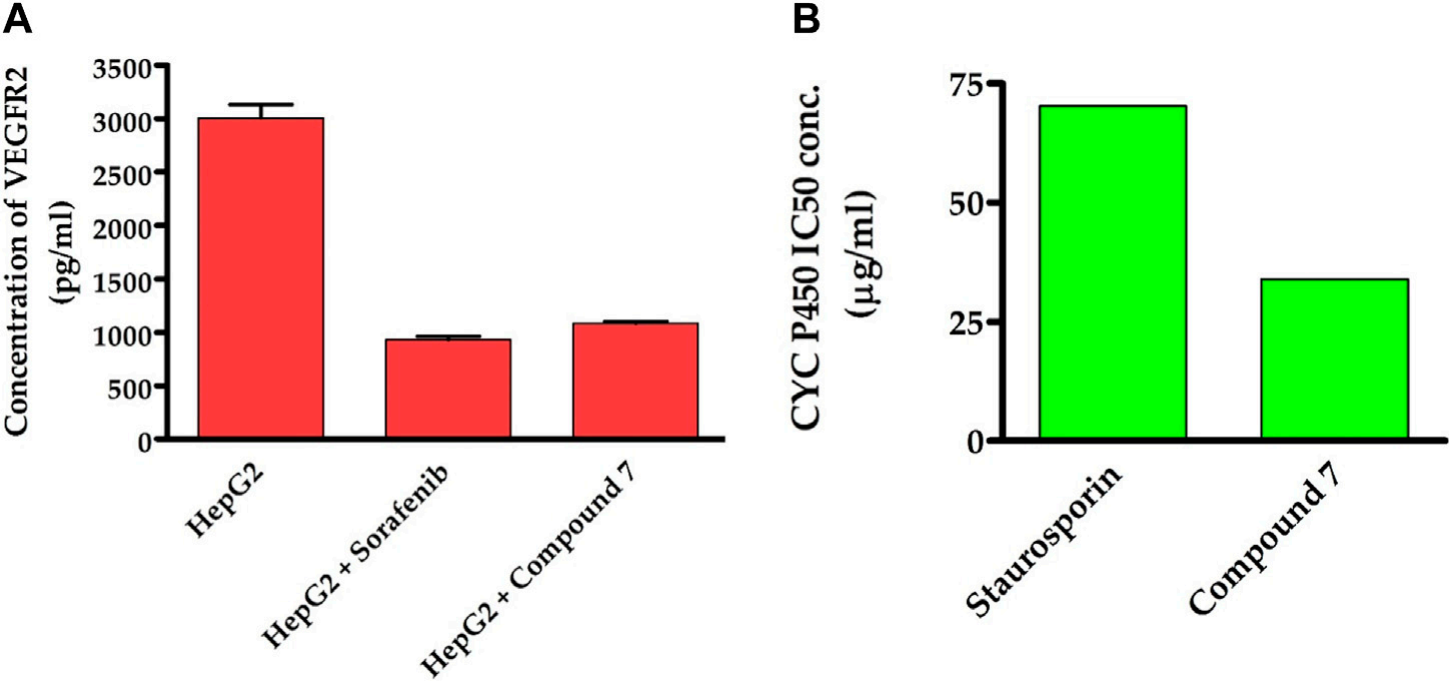
Dual-inhibitory activity of compound **7** toward VEGFR2 **(A)** and CYP450 **(B)** enzymes.

To obtain additional mechanistic insights into the antitumor activity of compound **7**, we investigated whether it has the ability to suppress the activity of VEGFR2 enzyme. As previously discussed, VEGFR-2 is a crucial target among angiogenesis-related kinases as it plays a key role in controlling cellular responses to VEGF in various cancer cells (Goel and Mercurio, 2013). Targeting VEGFR-2 signaling has become a key strategy in the search for novel drugs to treat numerous cancers that rely on angiogenesis (Liu et al., 2022). In recent years, the FDA has approved a variety of VEGFR-2 inhibitors as anti-angiogenic drugs for treating various solid tumors. Including sorafenib, sunitinib, and regorafenib (Ayala-Aguilera et al., 2022). Toward this, we were curious to explore whether the antitumor activity of compound **7** is linked to its ability to target the VEGFR-2 receptor inHep-G2 cells. As indicated in Figure 7, compound **7** exhibited a substantial inhibitory activity toward VEGFR2 protein (1,086 pg/mL), as compared to the untreated Hep-G2 cells (3,007 pg/mL). Interestingly, the inhibitory activity of compound **7** was similar to that of the reference sorafenib drug (932.4 pg/mL). The results suggest that the ability of compound **7** to target VEGFR2 protein activity may be responsible for its antitumor effects on Hep-G2 cells.

### 2.7 Assessment of CYP inhibitory activity

Our findings motivated us to delve deeper into the underlying mechanisms responsible for the potent antitumor activity of this class of compounds. CYP2D6 is a key enzyme primarily found in the liver that plays a critical role in metabolizing numerous drugs, including commonly prescribed medications and other xenobiotics. Its altered activity due to factors such as genetic variations may affect drug metabolism and efficacy, and it may interact with other liver enzymes. Recent studies suggest targeting CYP2D6 as a potential therapeutic approach for liver cancer (Peter Guengerich et al., 2016). To investigate whether the antiproliferative effect of compound **7** is linked to its capacity to inhibit CYP2D6 activity, we have extended our studies. In this regard, we have assessed the dose-dependent inhibitory behavior of compound **7** toward the CYP2D6 activity and utilized the standard drug staurosporin. As indicated in Figure 7, the results revealed that compound **7** possesses a significant and dose-dependent inhibitory feature toward CYP2D6 activity with IC_50_ of 34 μg/mL. Interestingly, compound **7** exhibited an inhibitory activity toward CYP2D6 which was 2.1-fold more potent than that of the standard staurosporin drug (70.29 μg/mL). These findings imply that the antitumor activity of compound **7** might be explained by its dual inhibitory activity toward both VEGFR2 and CYP2D6 proteins.

### 2.8 In silico computational studies

#### 2.8.1 *In silico* molecular modelling simulations

The technique of molecular docking simulation has been widely and effectively utilized to analyze how a bioactive ligand interacts with the active site of a specific protein, as well as to determine its binding score (Mohamed et al., 2021; Saied et al., 2021; Khirallah et al., 2022a; Mohamed et al., 2022a; Mohamed et al., 2022b; Healey et al., 2022). To further validate the potential of compound 7 as a dual-target inhibitor of VEGFR2 and CYP2D6 proteins, we employed *in silico* molecular docking analysis to evaluate its binding affinity towards the active sites of these proteins. Toward this, the crystal structure of the targeted proteins in complex with their co-crystallized ligand was accessed from protein data bank (access 1/2/2023, https://www.rcsb.org/); VEGFR2 protein (PDB code: *4asd*) and CYP2D6 protein (PDB code: *4xrz*) (McTigue et al., 2012; Brodney et al., 2015). The acquired crystal structures were first prepared for molecular simulation by removing extra chains and water and other extra molecules. The docking protocol was adapted to ensure that the co-crystallized molecule interacts in similar mode as reported in the crystal structure with low RMSD values.

##### 2.8.1.1 Molecular modelling simulation of compound **7** toward the active site of VEGFR2 protein

**FIGURE 8:**
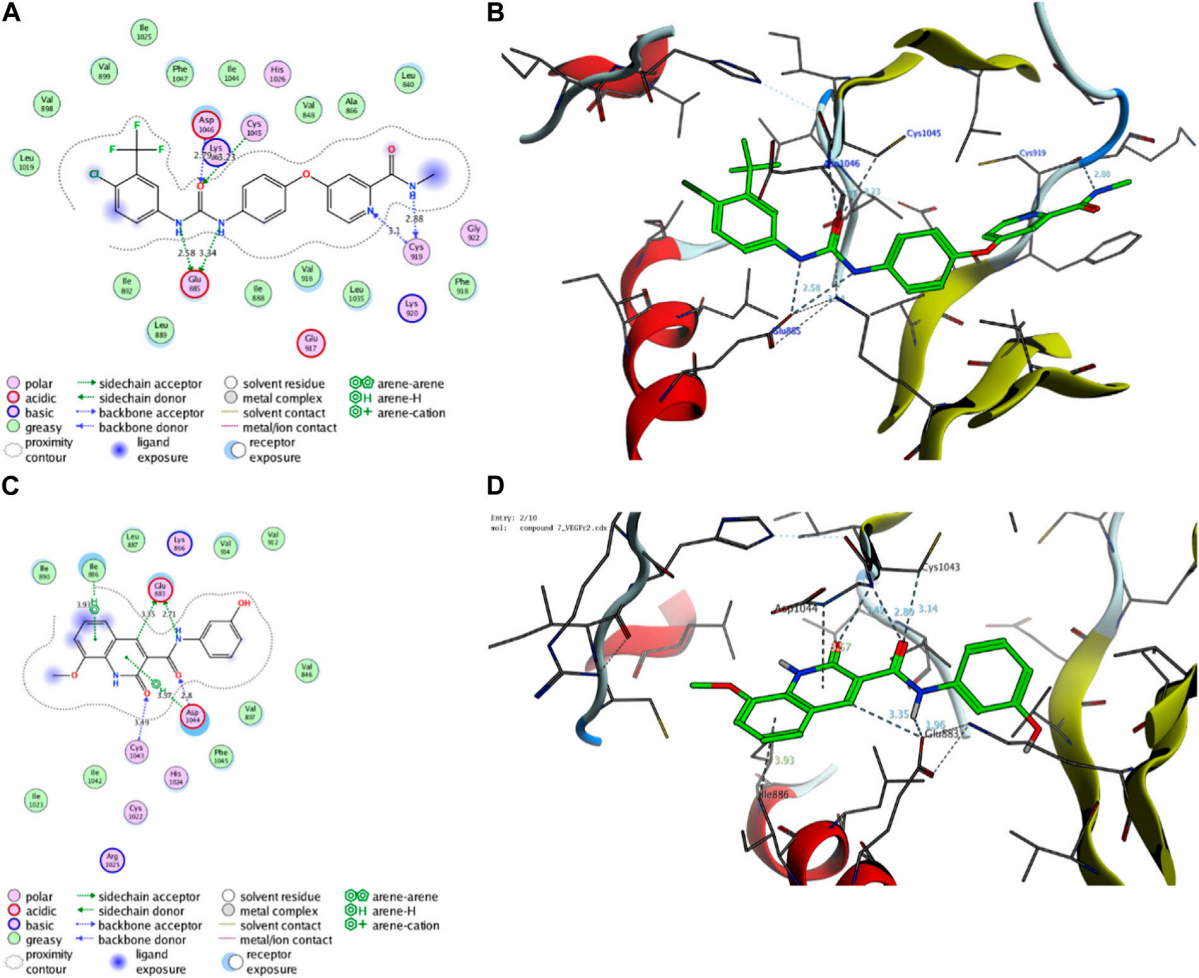
Representative binding modes of sorafenib **(A,B)** and compound **7 (C,D)** inside the active site of VEGFR2 (PDB code: *4asd*) protein through 2D and 3D molecular docking simulations.

Initially, we investigated the binding affinity and mode of interaction between compound **7** and the function pocket of the VEGFR2 protein. In our study, we utilized the reported VEGFR2-crystal structure in complex with sorafenib as a co-crystallized ligand (PDB code: *4asd*) (McTigue et al., 2012). We modified and adapted the applied protocol to ensure that sorafenib forms the main interactions reported in the complex structure in the binding pocket. As shown in Table 2; Figure 8, the redocking process of sorafenib drug into the active site of VEGFR2 protein revealed a set of H-bonding and hydrophobic interactions with several amino acid residues in the pocket with binding score of −15.29 kcal/mol. The urea moiety of sorafenib formed four H-bonding interactions with Glu885, Asp1046, and Cys1045 amino acid residues. Further, Cys919 residue in the active site could form a dual H-bonding interactions with the *N*-methylnicotinamide moiety. The bind of sorafenib was further supported by a network of hydrophobic interactions with a set of grassy amino acid residues in the active site (Figure 8).

**TABLE 2:** Binding score and interactions of compound 7, as compared to the co-crystallized ligand Sorafenib, toward the active cavity of VEGFR2 protein.

Protein (PDB code)	Compound	Binding score (kcal/mol)	Hydrophobic interactions		Hydrophilic interactions	Distance (A)
VEGFR2 (*4asd*)	Sorafenib	−15.29	Leu840	Val848	Cys919	3.10
			Ala866	Ile888	Cys919	2.88
			Leu889	Ile892	Cys1045	3.32
			Val898	Val899	Asp1046	2.79
			Val916	Phe918	Glu885	3.34
			Leu1019	Ile1025	Glu885	2.58
			Leu1035	Ile1044		
			Phe1047			
	**7**	−14.71	Val846	Ile886	Glu883	3.35
			Leu887	Ile890	Glu833	2.71
			Val897	Val912	Asp1044	2.80
			Val914	Ile1023	Asp1044	3.57
			Ile1042	Phe1045	Cys1043	3.49
					Ile886	3.93

On the other hand, compound **7** exhibited a considerable binding affinity toward the active site of VEGFR2 pocket with score of −14.71 kcal/mol. Analysis of the binding mode revealed that compound **7** has the ability to interact and form four H-bonding with Glu883 and Asp1044 amino acid residues through its 3-hydroxyphenyl-acetamide moiety. Further, the azacoumarin scaffold participated in the stability of conformer by forming H-bonding with Cys1043 residue and interacting with Ile886 residue through H-arene bonding (Figure 8). The binding of compound **7** was further supported by a network of hydrophobic amino acids in the pocket (Val846, Ile886, Leu887, Ile890, Val897, Val912, Val914, Ile1023, Ile1042, Phe1045). In agreement with our previous results, these findings affirm and explain the observed *in vitro* inhibitory activity of compound **7** toward VEGFR2 activity. Further, our results suggest that 8-methoxy-azacoumarin-3-carboxamide has the potential to serve as a primary structure for creating effective VEGFR2 inhibitors.

##### 2.8.1.2 Molecular modelling simulation of compound **7** toward the active site of CYP2D6 protein

**FIGURE 9:**
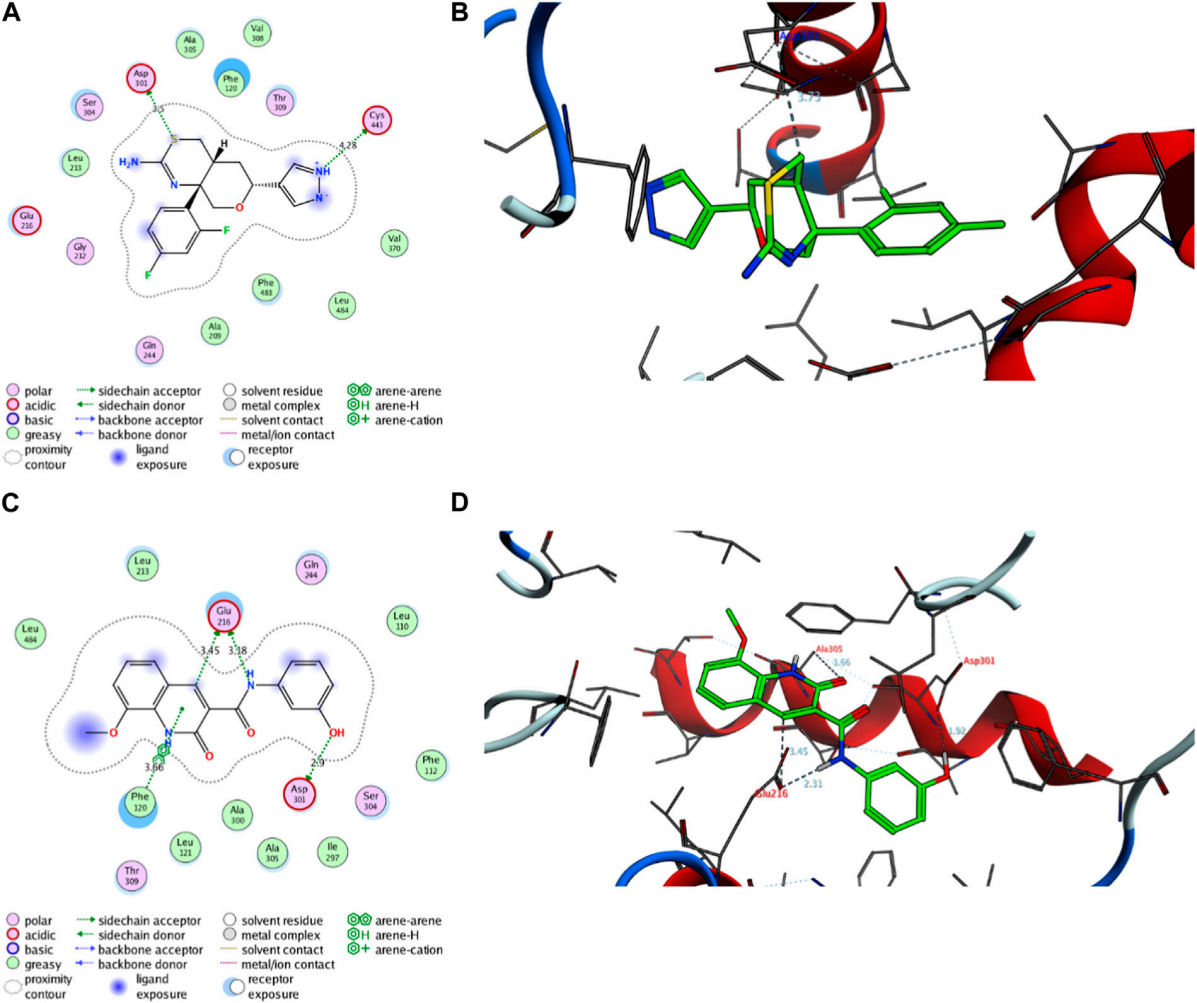
Representative binding modes of BACE1 Inhibitor 6 **(A,B)** and compound **7 (C,D)** inside the active site of CYP2D6 (PDB code: *4xrz*) protein through 2D and 3D molecular docking simulations.

Next, an evaluation was conducted to determine the binding affinity and mode of interaction between compound 7 and the active site of the CYP2D6 protein. Toward this, we initially validated the docking protocol by re-docking the co-crystallized ligand (BACE1 inhibitor 6) into the pocket of CYP2D6 protein and affirmed the mode of interaction and binding score, as compared to the reported data (PDB code: *4xrz*) (Brodney et al., 2015). Our analysis revealed that BACE1 inhibitor 6 possesses a significant binding affinity (−12.06 kcal/mol) and binds mainly to two amino acid residues (Asp301, and Cys443) by H-bonding interaction (Table 3). In addition, BACE1 inhibitor 6 interacts hydrophobically with several amino acids (Phe120, Ala209, Leu213, Ala305, Val308, Val370, Phe483, and Leu484) in the active site to assure the conformer stability (Figure 9).

**TABLE 3:** Binding score and interactions of compound 7, as compared to the co-crystallized ligand BACE1 inhibitor 6, toward the active cavity of CYP2D6 protein.

Protein (PDB code)	Ligand	Binding score (kcal/mol)	Hydrophobic interactions		Hydrophilic interactions	Distance (A)
CYP2D6 (*4xrz*)	**BACE1 Inhibitor 6**	−12.06	Phe120	Ala209	Asp301	3–50
			Leu213	Ala305	Cys443	4.28
			Val308	Val370		
			Phe483	Leu484		
	**7**	−12.97	Leu110	Phe112	Glu216	3.18
			Phe120	Leu121	Glu216	3.45
			Leu213	Ile297	Asp301	2.90
			Ala300	Ala305	Phe120	3.66
			Leu484			

Regarding compound **7**, the docking simulation analysis demonstrated that compound **7** displays a significant binding affinity toward the active site of CYP2D6 protein with binding score of −12.97 kcal/mol (Table 3). Evaluation of the binding mode revealed that compound **7**, in the most stable conformer, displays the ability to interact with several amino acid residues in the active site by forming a network of hydrophobic and H-bonding interactions. As shown in Figure 9, compound **7** bound through its azacoumarin scaffold to Glu216 and Phe120 amino acid residues by H-bonding and arene-arene interaction, respectively. Further, the amino group of the acetamide moiety could also form a strong H-bonding with Glu216 residue, suggesting the beneficial role of 3-carboxamide-substitution at the azacoumarin ring. This finding was further supported by the ability of phenolic-OH to form H-binding to Asp301 residue. Similarly, compound **7** showed the ability to form a set of hydrophobic interactions with Leu110, Phe112, Phe120, Leu121, Leu213, Ile297, Ala300, Ala305, Leu484 amino acid residues. Together, our molecular docking simulation studies explain and affirm the *in vitro* inhibitory activity of compound **7** toward the CYP2D6 protein and further demonstrate the dual-inhibitory activity of this compound toward VEGFR2 and CYP2D6 proteins.

#### 2.8.2 *In silico* toxicity and ADME prediction

Encouraged by our findings, we were interested in exploring the toxicity and drug likeness of the most active 8-methoxy-azacoumarin-3-carboxamide (compound **7**), as compared to the reference drugs sorafenib and staurosporine. Toward this, several computational analyses were *in silico* performed including SwissADME, Protox II, OSIRIS Property Explorer and pkCSM. As shown in Table 4, the analyses revealed that compound 7, sorafenib and staurosporine follow both of Lipinski rule and Veber rule without any violations (M.wt < 500 Da, TPSA<140 Å^2^, HBA<10, HBD<5). The ratio of hybridized C-sp3 atoms to the total carbon number (fraction of Csp^3^) indicated that both compound **7** and sorafenib exhibit a low saturation ratio (0.06–0.10), as compared to staurosporine (Csp^3^ = 0.32). Furthermore, the evaluation of solubility with LogS indicated that compound **7** exhibits soluble behavior with ESOL value of −3.27, while sorafenib and staurosporine showed moderately soluble characteristic with ESOL range of −5.06 to −5.11. The prediction of lipophilicity for the compounds was realized by MLOGP (Moriguchi octanol–water partition coefficient) and XLogP3 (partition coefficient) estimation. The analysis indicated that all tested compounds possess acceptable MLogP (1.61–2.60) and XLogP3 (1.99–3.24) values.

**TABLE 4:** *In silico* assessment of drug likeness, pharmacokinetics, and chemical properties of compound 7, sorafenib, and staurosporine.

Assessment	Parameter	Compound 7	Sorafenib	Staurosporine
Chemical properties	M.Wt (Da)	310.30	464.82	466.53
	TPSA (Å^2^)	91.42	92.35	69.45
	No. of H-bond acceptors	4	7	4
	No. of H-bond donors	3	3	2
	No. of rotatable bonds	4	9	2
	Fraction Csp3	0.06	0.10	0.32
Solubility	Log S (ESOL)	−3.27	−5.11	−5.06
	Solubility class	Soluble	Moderate	Moderate
Lipophilicity	Log *P*_o/w_ (XLogP3)	1.99	4.07	3.24
	Log *P*_o/w_ (MLogP)	1.61	2.91	2.60
Pharmacokinetics	skin permeation (Log *K*_p,_ cm/s)	−6.78	−6.25	−6.85
	BBB permeant	No	No	Yes
	GI absorption	High	Low	High
	P- glycoprotein substrate	No	No	Yes
	CYP2D6 inhibitor	Yes	Yes	Yes
Drug likeness	Drug-score*	0.86	0.2	0.37
	Bioavailability Score	0.55	0.55	0.55
	PAINS	0, alert	0, alert	0, alert
	Veber rule (violation)	Yes	Yes	Yes
	Lipinski rule (violation)	Yes	Yes	Yes
	Leadlikeness	Yes	No	No
	Synthetic accessibility	2.35	2.87	4.93

The assessment of drug likeness parameters indicated that all evaluated compounds exhibit a considerable bioavailability score (0.55) with no PAIN violation in their structures. Interestingly, compound **7** exhibited a high drug-score value (0.86), as compared to sorafenib and staurosporine (0.2 and 0.37, respectively). In addition, compound 7 showed accessibility as a leadlikeness structure with an easy synthetic approachability (2.35), as compared to sorafenib and staurosporine (2.87 and 4.93, respectively). In addition, the assessment of pharmacokinetic parameters showed that all compounds have no ability to pass the blood–brain barrier (BBB), while they, except for staurosporine, have a considerable permeability to gastrointestinal tract. Further, in agreement with our *in vitro* analysis, all compounds showed the ability to be inhibitors for CYP2D6 protein. Taken together, our *in silico* analysis indicates that compound **7** possesses a promising pharmacokinetic and drug likeness properties to be considered in the development of anticancer drugs.

The assessment of toxicity indicated that all compounds belong to class IV with an oral lethal dose for rats ranging from 2.077–2.464 mol/kg (Table 5). The maximum tolerated dose for humans was relatively low for sorafenib and staurosporine (0.253 and 0.271 log mg/kg/d, respectively), while compound 7 displayed a considerable higher dose (0.615 log mg/kg/d). Further, all tested compounds did not show features for AMES toxicity (except for staurosporine), and skin sensitization. The tested compounds did not display ability to inhibit human ether-a-go-go-related gene 1 (hERG 1), while they showed to be inhibitors for (hERG 2).

**TABLE 5:** *In silico* toxicity assessment for compound 7, sorafenib, and staurosporine.

Assessment parameter	Compound 7	Sorafenib	Staurosporine
Toxicity class	IV	IV	IV
Rat Acute Toxicity (LD_50_), mol/kg	2.077	2.14	2.464
AMES toxicity	No	No	Yes
Max. dose (human), (log mg/kg/d)	0.615	0.253	0.271
Skin Sensitization	No	No	No
hERG I inhibitor	No	No	No
hERG II inhibitor	Yes	Yes	Yes
Phosphoprotein p53, probability	Inactive, 0.96	Active, 0.57	Inactive, 0.68
Mitochondrial membrane potential, probability	Inactive, 0.70	Active, 0.79	Inactive, 0.70
Heat shock factor response element, probability	Inactive, 0.88	Inactive, 0.96	Inactive, 0.94
Immunotoxicity, probability	Active, 0.96	Active, 0.92	Active, 0.92
Carcinogenicity, probability	Inactive, 0.62	Inactive, 0.50	Inactive, 0.61
Hepatotoxicity, probability	Active, 0.69	Active, 0.82	Inactive, 0.73
Cytotoxicity, probability	Inactive, 0.93	Active, 0.77	Active, 0.79
Mutagenicity, probability	Inactive, 0.97	Inactive, 0.79	Inactive, 0.52

As indicated in Table 5, all tested compounds displayed inactivity toward carcinogenicity mutagenicity, and heat shock factor response element with high probability values. The analysis further revealed that all tested compounds, except for sorafenib, exhibit no activity toward tumor suppressor phosphoprotein p53, and mitochondrial membrane potential with probability values ranging from 0.68–0.96. On the other hand, all compounds displayed considerable probability values toward immunotoxicity and hepatotoxicity (except for staurosporine). Interestingly, compound 7 showed inactivity to cytotoxicity with high probability value (0.93), while sorafenib and staurosporine exhibited activity to cytotoxicity with considerable probability. In conclusion, considering the toxicity profile observed, it can be inferred that compound **7** displayed a favorable safety profile. This profile was characterized by its non-carcinogenic, non-mutagenic, and non-cytotoxic properties, along with a satisfactory LD_50_ value.

**FIGURE 10:**
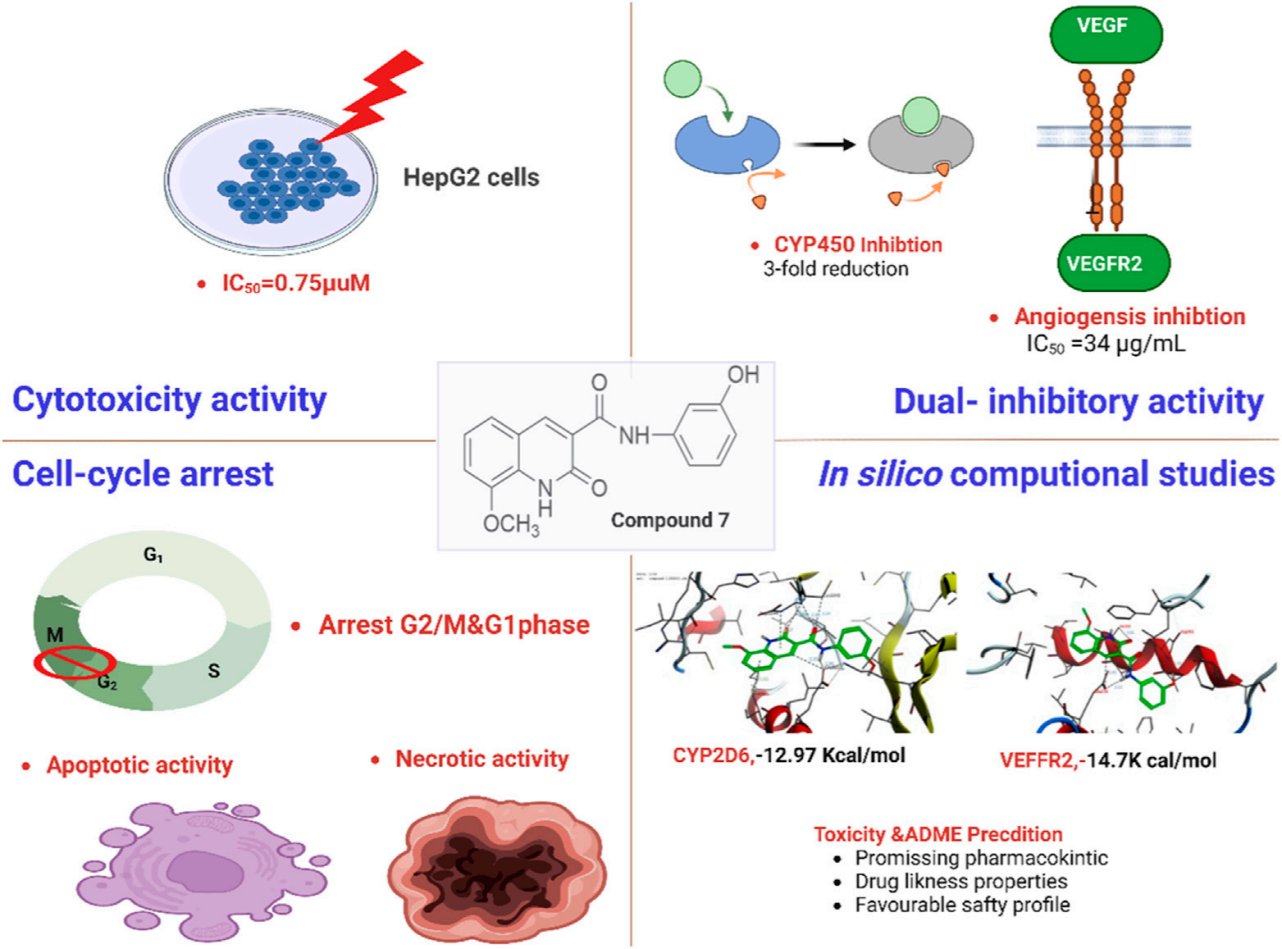
Representative graph for the antitumor potential of compound **7** against liver cancer.

Taken together, our findings indicate that compound **7** demonstrates extraordinary cytotoxic properties against liver cancer cells by interfering with DNA replication, initiating programmed cell death, and displaying dual inhibitory activity against VEGFR-2 and CYP450 enzymes, showcasing its effectiveness. Further, *in silico* pharmacokinetic analysis indicated that compound **7** possesses promising pharmacokinetic and drug-likeness properties to be considered in the development of anticancer drugs (Figure 10). Therefore, the presented findings indicate that compound **7** could be a potential lead compound for the further development of potent anti-liver cancer agents.

## 3 Materials and methods

### 3.1 General information (instruments, analysis, and reagents)

All the chemicals and solvents employed in this study were acquired from reputable commercial sources and possessed a high level of purity, meeting analytical grade standards. Rigorous quality control protocols were meticulously implemented to guarantee the precision and reproducibility of the experimental data. Proper calibration of instruments, careful sample preparation, and appropriate reference standards were used to ensure reliable and accurate results in the characterization and analysis of the compounds. The 1H and 13C nuclear magnetic resonance (NMR) spectra were recorded on a Bruker avance 400 MHz spectrometer for ^1^H NMR and 100 MHz spectrometer for ^13^C NMR, respectively, using DMSO-d_6_ solvent containing tetramethylsilane as an internal standard. The NMR measurements allowed for the characterization and structural elucidation of the compounds by analyzing the chemical shifts in δ (parts per million). Microanalytical data, including the determination of carbon, hydrogen, and nitrogen content, were obtained using a Perkin-Elmer 2,400 series CHN analyzer, which provides accurate elemental analysis and helps in determining the molecular formula of the compounds. The electron ionization mass spectrometry (EI-MS) was carried out using an Agilent Technologies 6890N gas chromatograph (GC) with a selective detector 5,973 mass spectrometer. The EI-MS analysis provided information about the mass-to-charge ratio of the compounds, allowing for the identification and confirmation of their molecular weights and molecular ions. Melting points of crystalline compounds were measured using an electrothermal melting point apparatus without any correction applied. The melting point, which is the temperature at which a solid compound changes from a solid to a liquid state, is a critical physical property that can provide insights into the purity, crystallinity, and identity of the compounds. The melting point data were carefully recorded and compared with literature values or reference standards to confirm the identity and purity of the compounds. Infrared (IR) spectra were recorded using a Bruker FT-8000 spectrometer, which allows for the analysis of molecular vibrations and functional groups in the infrared region. The IR spectra provides information about the presence of various chemical bonds and functional groups in the compounds, helping in the identification and characterization of the compounds.

### 3.2 Synthetic protocols and analytical assessments

#### 3.2.1 Synthesis of ethyl 8-methoxycoumarin-3-carboxylate (**1**)

Compound **1** was obtained by fusion of a mixture of 3-methoxy-2-hydroxybenzaldehyde (0.01 mol) and diethylmalonate (0.01 mol) on a hot-plate in the presence of piperidine (1 mL) for 5 min. Subsequently, ethanol (20 mL) was inserted to the reaction mixture and the resulting mixture was heated for 2 h under reflux. After the TLC analysis demonstrated a complete reaction, the reaction mixture was cooled, and poured into ice-water while stirring. The mixture was then neutralized with dilute HCl (2%), and the resulting solid was collected with filtration. The obtained crude solid was washed with water and dried. Finally, the product was re-crystallized from ethanol to give **2** as colorless crystals. Yield 86%, m.p. 105°C, IR (KBr) υ_max_: 1735, 1715 (C=O), 1,605, 1,583 (C=C), 1,125, 1,083 (C-O) cm^−1^.^1^H-NMR (DMSO-d_6_, ppm) *δ*: 1.32 (t, 3H, CH_3_), 3.92 (s, 3H, OCH_3_), 4.31 (q, 2H, OCH_2_), 7.30–7.45 (m, 3H, Ar-H), 8.71 (s, 1H, H-4 of coumarin ring). ^13^C-NMR (DMSO-d_6_, ppm) *δ*: 164.45, 163.04 (C=O), 157.15, 154.41 (C-O), 149.14 (C-4 of pyranone ring), 134.95, 130.73, 125.30, 118.76, 118.24, 116.60 (C-aromatic and C-3 of pyranone ring), 61.72 (OCH_2_), 57.10 (OCH_3_), 14.53 (CH_3_). Anal. Calcd for C_13_H_12_O_5_ (M. wt. = 248): C, 62.90; H, 4.84. Found: C, 62.61; H, 4.54.

#### 3.2.2 Synthesis of 8-methoxycoumarin-3-carboxylic acid (**2**)

A solution containing (1, 0.01 mol) of coumarin ester dissolved in 25 mL of ethanoic acid was subjected to a reaction with HCl (4N, 20 mL). The resulting mixture was heated under reflux for 2 h, and the progress of the reaction was monitored using TLC analysis. Once complete hydrolysis had occurred, the reaction mixture was carefully poured into crushed ice and left at room temperature for 18 h. The solid product that formed was separated by filtration, thoroughly washed with water, and subsequently dried. Recrystallization of the crude product utilizing ethanol as a solvent afforded compound 2 as colorless crystals. Yield 57%, m.p. 186 °C. IR (KBr) υ_max_: 3,355–2,951 (br. OH), 1726–1703 (br. C=O), 1,610, 1,580 (C=C), 1,036, 1,025 (C-O) cm^−1^.^1^H-NMR ((DMSO-d_6_, ppm) *δ*: 3.93 (s, 3H, OCH_3_), 7.31–7.45 (m, 3H, Ar-H), 8.71 (s, 1H, H-4 of coumarin ring). ^13^C-NMR (DMSO-d_6_, ppm) *δ*: 164.49, 156.90 (C=O), 148.95, 146.67 (C-O), 144.24 (C-4 of coumarin ring), 125.18, 121.52, 119.07, 118.96, 116.61 (C-aromatic and C-3 of pyranone ring), 56.60 (OCH_3_). MS: *m/z* (%) = 221 (M^+^+1, 16.00), 220 (M^+^, 96.36), 203 (22.34), 177 (8.93), 176 (100), 161 (15.58), 149 (7.22), 148 (38.72), 147 (20.17), 146 (6.35), 141 (7.33), 139 (34.97), 133 (61.14), 131 (4.08), 120 (17.92), 119 (12.91), 118 (18.20), 117 (2.41), 111 (7.98), 105 (52.55), 104 (7.43), 103 (12.87), 102 (10.13), 91 (17.12), 90 (12.72), 89 (24.94), 88 (5.49), 77 (33.45), 76 (16.34), 75 (7.56), 65 (7.99), 63 (5.95), 62 (1.90), 51 (13.66). Anal. Calcd for C_11_H_8_O_5_ (M. wt. = 220): C, 60.00; H, 3.64. Found: C, 59.81; H, 3.33.

#### 3.2.3 Synthesis of *N*-(3-hydroxy)-phenyl 8-methoxycoumarin-3-carboxamide (**3**)

Treatment of coumarin-3-carboxlic acid (**2**, 0.01 mol) with thionyl chloride (25 mL) afforded a clear solution which was subsequently heated for 2 h under reflux. After excess thionyl chloride was removed under reduced pressure, the resulting mixture was treated with 3-aminophenol (0.01 mol) and dimethyl formamide (20 mL). The obtained reaction mixture was subjected to reflux conditions for an additional 2 h, during which it was heated. The reflux continued until the TLC analysis confirmed that the reaction had fully completed. Following this, the reaction mixture was cooled to room temperature and carefully introduced into ice-water while maintaining continuous stirring. The resulting mixture was allowed to stand overnight at ambient temperature. The afforded crude solid was collected by filtration, washed with water, and dried. Purification of obtained crude product by recrystallization from ethanol as a solvent provided compound **3** as yellow crystals. Yield 71%, m.p. 256 °C. IR (KBr) υ_max_: 3,410 (OH), 3,225 (NH), 1725–1,690 (br. C=O), 1,610, 1,580 (C=C), 1,121, 1,081, 1,035 (C-O) cm^−1^.^1^H-NMR (DMSO-d_6_, ppm) *δ*: 3.96 (s, 3H, OCH_3_), 6.57–7.54 (m, 7H, Ar-H), 8.87 (s, 1H, H-4 of pyranone ring), 9.60 (s, 1H, OH), 10.59 (s, 1H, NH) ppm. ^13^C-NMR (DMSO-d_6_, ppm) *δ*: 160.72, 160.14 (C=O), 158.28, 147.97, 146.78 (C-O), 143.61 (C-4 of pyranone ring), 139.38, 130.25, 125.72, 121.63, 120.60, 119.53, 116.64, 111.94, 111.00, 107.32 (C-aromatic and C-3 of pyranone ring), 56.70 (OCH_3_) ppm. MS: *m/z* (%) = 311 (M^+^, 7.38), 310 (M^+^-1, 1.34), 283 (2.12), 249 (11.46), 248 (95.82), 247 (6.97), 220 (1.08), 204 (9.87), 203 (45.49), 202 (64.10), 192 (1.54), 188 (1.34), 177 (9.93), 176 (100), 175 (14.60), 161 (1.76), 160 (1.43), 148 (7.16), 147 (3.32), 146 (2.22), 133 (7.17), 120 (5.39), 119 (21.69), 118 (7.91), 117 (12.18), 116 (3.07), 115 (3.56), 105 (26.27), 104 (9.63), 103 (3.69), 91 (11.09), 90 (4.29), 89 (16.53), 88 (10.24), 77 (22.00), 76 (28.07), 75 (9.02), 65 (7.02), 63 (8.37), 62 (3.50), 51 (5.96), 50 (9.40). Anal. Calcd for C_17_H_13_NO_5_ (M. wt. = 311): C, 65.59; H, 4.18; N, 4.50. Found: C, 65.33; H, 3.89; N, 4.22.

#### 3.2.4 General procedure for the halogenation reactions (formation of compounds **4** and **8**)

A solution containing 0.01 mol of either Compound **3** or Compound **7** was prepared by dissolving it in 20 mL of glacial acetic acid at ambient temperature. The resulting solution was then gradually treated with a bromine solution (0.01 mol) in 15 mL of glacial acetic acid through dropwise addition. After the reaction mixture was placed under reflux at 60°C, it was allowed to stir for 20 min, the characteristic color of bromine faded away, giving rise to the formation of a solution with a distinct yellow color. Subsequently, at room temperature, a new aliquot of bromine-ethanoic acid solution (1 mL) was gradually introduced to the reaction mixture, followed by stirring for an additional duration of 45–60 min. Once the TLC analysis confirmed that the reaction had reached completion, the reaction mixture was carefully transferred into ice-water while vigorously stirring. The resulting solid product was subsequently collected through filtration. The crude solid was finally washed with water, dried, and purified by recrystallization utilizing a proper solvent to furnish the desired compound **4** or **8**.

##### 3.2.4.1 Synthesis of N-(3-hydroxy)phenyl 5-bromo-8-methoxycoumarin-3-carboxamide (**4**)

The entitled compound was synthesized following the abovementioned procedure to provide compound **4** as yellow crystals after recrystallization using ethanol as a solvent. Yield 63%, m.p. 289°C. IR (KBr) υ_max_: 3,420 (br. OH), 3,218 (br. NH_2_), 1725–1,695 (br. C=O), 1,605, 1,588 (C=C), 1,087, 1,031 (C-O) cm^−1^.^1^H-NMR and ^13^C-NMR: no data because compound **4** was insoluble in the available solvent. MS: *m/z* (%) = 389 (M^+^, unstable), 348 (2.23), 346 (3.66), 328 (21.03), 327 (1.44), 326 (20.14), 283 (8.30), 281 (6.72), 257 (2.64), 256 (12.79), 255 (2.54), 254 (11.80), 219 (6.33), 213 (5.05), 211 (2.13), 199 (6.90), 197 (8.45), 191 (4.86), 185 (3.68), 183 (5.18), 175 (6.34), 173 (2.71), 169 (6.55), 168 (2.42), 167 (5.34), 162 (21.60), 161 (10.57), 160 (28.21), 158 (14.23), 157 (8.58), 152 (10.37), 147 (12.72), 137 (8.60), 135 (5.03), 134 (3.79), 133 (6.74), 131 (4.62), 129 (10.63), 127 (2.91), 119 (10.63), 117 (6.85), 111 (12.25), 110 (9.70), 109 (8.37), 107 (5.02), 105 (29.52), 103 (30.71), 98 (16.26), 97 (25.78), 96 (9.50), 95 (36.29), 91 (21.71), 89 (7.52), 88 (12.00), 87 (11.72), 86 (53.76), 85 (40.86), 84 (100), 83 (42.82), 82 (22.22), 81 (38.44), 79 (22.44), 77 (28.80), 76 (9.01), 75 (37.90), 74 (21.29), 73 (24.16), 71 (43.36), 70 (18.86), 69 (71.40), 67 (16.58), 65 (3.53), 63 (8.64), 60 (19.13), 57 (70.84), 56 (18.66), 55 (57.24), 51 (38.23), 50 (11.91). Anal. Calcd for C_17_H_12_BrNO_5_ (M. wt. = 389): C, 52.44; H, 3.08; N, 3.60. Found: C, 52.18; H, 2.96; N, 3.33.

##### 3.2.4.2 Synthesis of N-(3-hydroxy)phenyl 5-bromo-8-methoxy-azacoumarin-3-carboxamide (**8**)

The entitled compound **8** was obtained as yellow crystals following the abovementioned procedure after recrystallization using ethanol as a solvent. Yield 64%, m.p. 255°C. IR (KBr) υ_max_: 3,421 (OH), 3,221 (br. NH), 1705, 1,693 (C=O), 1,605, 1,583 (C=C), 1,117, 1,063 (C-O) cm^−1^.^1^H-NMR ((DMSO-d_6_, ppm) *δ*: 3.96 (s, 3H, OCH_3_), 7.06–7.98 (m, 7H, Ar-H and NH), 8.80 (s, 1H, H-4 of pyridinone ring), 10.38, 10.39 (s, 1H, NH of two isomers) ppm. ^13^C-NMR (DMSO-d_6_, ppm) *δ*: 160.39, 160.15, 159.71, 159.51 (C=O), 151.88, 146.74 (C-O and C-N), 144.98 (C-4 of azacoumarin ring), 136.07, 135.92, 134.22, 129.10, 125.79, 121.87, 120.23, 119.32, 118.88, 118.45, 115.39, 113.56, 112.80, 111.80 (C-aromatic and C-3 of azacoumarin of two isomers), 57.03, 56.76 (COCH_3_ of two isomers) ppm. MS: *m/z* (%) = 390 (M^+^+2, 2.68), 389 (M^+^+1, 0.55), 388 (M^+^, 1.20), 312 (1.01), 311 (20.55), 310 (1.99), 283 (0.12), 282 (3.66), 281 (2.43), 256 (2.51), 213 (1.62), 212 (1.12), 211 (1.12), 204 (14.93), 203 (74.99), 202 (100), 167 (14.24), 157 (1.14), 149 (53.52), 133 (2.43), 132 (1.61), 131 (1.91), 121 (1.90), 119 (16.31), 117 (3.23), 116 (3.88), 115 (1.93), 113 (5.05), 111 (6.42), 109 (3.02), 104 (3.10), 103 (2.20), 97 (13.60), 96 (32.38), 95 (11.09), 94 (23.27), 93 (9.77), 91 (6.42), 89 (9.62), 85 (12.08), 83 (24.20), 82 (21.35), 81 (31.25), 80 (18.10), 79 (13.90), 77 (12.47), 76 (23.50), 75 (7.95), 74 (10.01), 73 (33.71), 71 (41.11), 70 (23.99), 69 (49.86), 68 (12.51), 65 (8.58), 63 (5.44), 62 (3.72), 60 (20.51), 57 (59.16), 56 (21.39), 55 (46.43), 51 (2.84), 50 (3.25). Anal. Calcd for C_17_H_13_BrN_2_O_4_ (M. wt. = 388): C, 52.58; H, 3.35; N, 7.22. Found: C, 52.33; H, 3.18; N, 7.07.

#### 3.2.5 General procedure for the synthesis of acetoxy derivatives **5** and **6**

Compound **3** or Compound **4** (0.01 mol) was reacted with 20 mL of acetic anhydride, and the resulting mixture was refluxed for 2 h. After stirring for an additional 2 h under the same conditions, the reaction was stopped by adding it to an ice-water mixture. The resulting reaction mixture was allowed to settle for 24 h at room temperature, and the precipitate formed was separated, rinsed with water, and dried. The crude final products were subjected to recrystallization step utilizing a suitable solvent to afford the desired product (compounds **5** and **6**).

##### 3.2.5.1 Synthesis of N-(2-acetoxy)phenyl 8-methoxycoumarin-3-carboxamide (**5**)

The entitled compound was obtained as yellow crystals following the abovementioned protocol after recrystallization from ethanol. Yield 62%, m.p. 184 °C. IR (KBr) υ_max_: 3,225 (NH), 1746, 1728, 1,698 (C=O), 1,605, 1,588 (C=C), 1,121, 1,078, 1,038 (C-O) cm^−1^.^1^H-NMR ((DMSO-d_6_, ppm) *δ*: 2.30 (s, 3H, COCH_3_), 3.97 (s, 3H, OCH_3_), 6.92–7.67 (m, 7H, Ar-H), 8.88 (s, 1H, H-4 of coumarin ring), 10.77 (s, 1H, NH) ppm. ^13^C-NMR (DMSO-d_6_, ppm) *δ*: 169.67, 160.62, 160.49 (C=O), 151.24, 148.09, 146.81 (C-O), 143.66 (C-4 of coumarin ring), 139.37, 130.33, 125.76, 121.67, 120.61, 119.47, 118.15, 117.63, 116.76, 113.86 (C-aromatic and C-3 of coumarin ring), 56.73 (OCH_3_), 21.35 (COCH_3_) ppm. MS: *m/z* (%) = 353 (M^+^, 9.25), 312 (5.14), 311 (30.57), 310 (10.92), 294 (1.73), 283 (19.16), 282 (4.74), 204 (9.94), 203 (100), 202 (26.96), 175 (2.08), 160 (1.19), 144 (2.31), 120 (1.81), 119 (28.94), 118 (4.03), 117 (19.12), 116 (12.54), 110 (1.24), 109 (10.30), 105 (7.49), 104 (8.01), 103 (3.21), 101 (3.33), 91 (6.37), 90 (2.97), 89 (20.78), 88 (4.76), 83 (3.19), 80 (1.86), 79 (3.40), 77 (17.65), 76 (21.01), 75 (4.90), 65 (10.16), 64 (3.25), 63 (8.47), 57 (7.75), 56 (2.76), 55 (6.31), 51 (7.34). Anal. Calcd for C_19_H_15_NO_6_ (M. wt. = 353): C, 64.59; H, 4.25; N, 3.96. Found: C, 64.33; H, 4.04; N, 3.68.

##### 3.2.5.2 Synthesis of N-(3-acetoxy)phenyl 5-bromo-8-methoxycoumarin-3-carboxamide (**6**)

The desired compound was obtained as pale-yellow crystals following the abovementioned protocol after recrystallization utilizing ethanol as a solvent. Yield 67%, m.p. 205 °C. IR (KBr) υ_max_: 3,222 (NH), 1749, 1723, 1,691 (C=O), 1,610, 1,589 (C=C), 1,121, 1,083, 1,027 (C-O) cm^−1^.^1^H-NMR ((DMSO-d_6_, ppm) *δ*: 2.38, 2.44 (s, 3H, COCH_3_ of two isomers), 3.96 (s, 3H, OCH_3_), 7.37–8.45 (m, 6H, Ar-H), 8.99, 9.01 (s, 1H, H-4 of coumarin of two isomers), 11.19, 11.30 (s, 1H, NH of two isomers) ppm. ^13^C-NMR (DMSO-d_6_, ppm) *δ*: 168.64, 161.39, 160.42 (C=O), 149.94, 147.94, 146.80 (C-O), 143.84 (C-4 of coumarin ring), 136.92, 135.97, 125.90, 122.06, 119.54, 118.47, 117.90, 117.70, 117.34, 117.14, 111.39, 111.17 (C-aromatic and C-4 of coumarin ring), 56.90, 56.79 (OCH_3_ of two isomers), 20.98, 20.71 (COCH_3_ of two isomers) ppm. MS: *m/z* (%) = 431 (M^+^, unstable), 390 (5.25), 389 (3.09), 388 (5.93), 350 (11.40), 349 (4.51), 348 (36.89), 347 (11.70), 346 (40.57), 345 (9.86), 344 (12.37), 329 (13.70), 328 (98.51), 327 (26.31), 326 (52.57), 325 (64.80), 311 (9.49), 310 (3.09), 308 (1.50), 300 (3.41), 299 (2.87), 298 (5.58), 284 (3.27), 283 (49.71), 282 (14.45), 281 (46.58), 280 (7.54), 279 (3.65), 266 (2.45), 265 (1.59), 257 (59.69), 256 (59.69), 255 (23.44), 254 (31.31), 253 (47.79), 247 (8.86), 241 (4.19), 240 (3.60), 239 (9.86), 238 (2.99), 228 (6.33), 227 (3.87), 226 (7.09), 219 (24.54), 218 (7.07), 213 (9.13), 212 (4.53), 211 (13.74), 210 (3.75), 204 (3.14), 203 (41.84), 201 (3.74), 199 (16.31), 198 (4.61), 197 (19.61), 196 (2.77), 191 (11.35), 185 (9.45), 184 (3.55), 183 (8.88), 176 (3.08), 175 (15.90), 174 (3.77), 173 (8.81), 169 (10.80), 168 (3.18), 167 (18.34), 157 (8.74), 156 (8.94), 155 (7.12), 154 (6.52), 153 (5.99), 149 (21.30), 147 (6.61), 141 (4.04), 132 (5.77), 131 (7.36), 129 (4.00), 120 (5.96), 119 (14.24), 118 (8.41), 117 (13.97), 116 (4.57), 105 (6.05), 104 (11.41), 103 (49.99), 108 (9.96), 101 (7.64), 98 (3.85), 97 (11.62), 96 (6.12), 95 (7.34), 93 (5.75), 92 (5.72), 91 (14.41), 90 (19.51), 89 (25.94), 88 (21.28), 87 (29.66), 86 (14.27), 85 (16.58), 84 (13.10), 83 (15.95), 82 (14.14), 81 (21.73), 80 (11.27), 79 (18.76), 78 (12.60), 77 (29.91), 76 (36.39), 75 (100), 74 (74.60), 73 (20.19), 69 (25.38), 65 (9.45), 63 (23.34), 62 (20.53), 61 (15.90), 60 (15.02), 57 (32.74), 55 (31.69), 53 (22.11), 51 (12.14), 50 (12.23). Anal. Calcd for C_19_H_14_BrNO_6_ (M. wt. = 431): C, 52.90; H, 3.25; N, 3.25. Found: C, 52.73; H, 3.01; N, 3.11.

#### 3.2.6 Synthesis of N-(3-hydroxy)phenyl 8-methoxy-azacoumarin-3-carboxamide (**7**)

Compound **3** (0.01 mol) was dissolved in 40 ml of absolute ethanol, and then reacted with 0.03 mol of anhydrous potassium carbonate. Before adding the ammonia solution (35%, 10 mL), the prepared liquid was allowed to heat through reflux for 30 min. The resulting reaction mixture was left to reflux for a further 4 h while TLC analysis monitored the reaction’s development. After TLC analysis indicated a complete reaction, the mixture was cooled to room temperature and then carefully transferred into an ice-water mixture to rapidly halt the reaction. The obtained mixture was then carefully neutralized with dilute HCl (2%) till pH∼7. the solid obtained from the reaction was isolated through filtration, rinsed with water, and dried. The crude product obtained was then subjected to recrystallization using ethanol as the solvent, resulting in the formation of yellow crystals of compound **7**. Yield 63%, m.p. 225 °C. IR (KBr) υ_max_: 3,428 (br. OH), 3,228 (NH), 1705–1,695 (br. C=O), 1,605, 1890 (C=C), 1,093, 1,063, 1,032 (C-O) cm^−1^.^1^H-NMR (DMSO-d_6_, ppm) *δ*: 3.96 (s, 3H, OCH_3_), 6.56–7.55 (m, 7H, Ar-H), 8.87 (s, 1H, H-4 of coumarin ring), 9.60 (s, 1H, OH), 10.60 (s, 1H, NH) ppm. ^13^C-NMR (DMSO-d_6_, ppm) *δ*: 160.73, 160.12 (C=O), 158.29, 147.98, 146.78 (C-O), 143.61 (C-4 of azacoumarin ring), 139.38, 130.25, 125.71, 121.63, 120.57, 119.52, 116.63, 111.94, 111.00, 107.32 (C-aromatic and C-3 of pyridinone ring), 56.70, 56.51 (OCH_3_ of two isomers). MS: *m/z* (%) = 310 (M^+^, 1.18), 283 (4.83), 282 (1.39), 250 (1.02), 243 (5.97), 242 (2.76), 227 (4.30), 226 (5.74), 220 (2.63), 219 (23.78), 212 (2.18), 211 (1.36), 205 (2.18), 204 (9.74), 203 (60.89), 202 (3.26), 201 (1.13), 200 (1.04), 196 (1.09), 183 (1.62), 178 (2.85), 177 (12.73), 176 (19.36), 175 (6.98), 174 (2.71), 173 (2.36), 172 (2.49), 162 (2.84), 161 (3.71), 160 (1.50), 155 (2.14), 154 (2.79), 152 (1.12), 151 (2.97), 150 (8.62), 149 (2.90), 148 (10.18), 147 (7.00), 146 (3.68), 145 (2.35), 144 (2.34), 137 (4.92), 136 (13.70), 135 (12.16), 134 (5.76), 133 (17.64), 131 (2.47), 130 (1.79), 128 (1.46), 127 (2.38), 124 (10.66), 123 (2.20), 122 (5.39), 121 (4.42), 120 (8.99), 119 (14.22), 118 (8.09), 117 (9.10), 116 (6.37), 115 (4.04), 111 (1.30), 110 (8.19), 109 (100), 108 (12.86), 107 (10.10), 106 (8.98), 105 (22.58), 104 (10.45), 103 (6.88), 102 (6.27), 101 (4.07), 98 (1.71), 94 (3.59), 93 (6.52), 92 (6.79), 91 (17.02), 90 (8.86), 89 (26.52), 88 (6.74), 87 (4.26), 85 (3.85), 84 (2.71), 82 (4.90), 81 (31.30), 80 (46.18), 79 (11.60), 78 (11.49), 77 (37.03), 76 (29.15), 75 (11.74), 72 (3.15), 69 (3.74), 68 (5.75), 67 (2.65), 66 (5.81), 65 (25.88), 64 (10.80), 63 (23.86), 62 (12.46), 61 (3.81), 57 (2.45), 55 (7.37), 54 (7.86), 53 (22.56), 52 (17.37), 51 (27.99), 50 (14.41). Anal. Calcd for C_17_H_14_N_2_O_4_ (M. wt. = 310): C, 65.80; H, 4.55; N, 9.03. Found: C, 65.64; H, 4.29; N, 8.89.

### 3.3 Cell viability assay

HepG2 and HL-7702 cells were seeded in a 96-well plate at a density of 1 × 104 cells/well. The plate was subsequently placed in a humidified incubator with 5% CO2 at 37°C for 48 h to allow the cells to adhere and proliferate. Following this initial incubation period, the cells were exposed to varying concentrations of the synthesized compounds. The concentrations may have ranged from a lower to higher dose, depending on the experimental design. The molecules were dissolved in an appropriate solvent, DMSO, to obtain the desired concentrations. Control wells with no drug treatment were also included. Following the addition of the molecules, the plate was further incubated for 24 h at 37°C to allow the cells to respond to the drug treatment. The duration of the incubation may have varied depending on the specific experimental requirements. At the end of the 24-h treatment, (3-(4,5-dimethylthiazol-2-yl)-2,5-diphenyltetrazolium bromide) MTT dye was added to each well. MTT is a yellow tetrazolium salt that is utilized by metabolically active cells and converted into purple formazan crystals by mitochondrial enzymes in viable cells. After a further incubation of 4 h at 37°C, the medium in each well was carefully aspirated, and 100 μL of DMSO was added to dissolve the purple formazan crystals formed by viable cells. DMSO is a common solvent used to dissolve the formazan crystals and extract the intracellular purple product. The plate was then subjected to colorimetric measurement using an ELISA plate reader at a wavelength of 570 nm. The intensity of the purple color is proportional to the number of viable cells and reflects the growth condition of the cells in each well. The results were analyzed by calculating the percentage of cell viability or cell growth inhibition compared to the control wells. The data were typically presented as a dose-response curve, and the concentration of the drug that caused a 50% inhibition of cell growth (IC50) was determined as a measure of the drug’s potency. The experiments were performed with at least three replicates for each concentration, and the entire experiment was repeated at least three times to ensure the reliability of the results. Statistical analysis may have been performed to determine the significance of differences between the treated groups and the control group, using appropriate statistical tests.

### 3.4 DNA flow cytometry assay

HepG-2 cells were seeded in 96-well plates at a density of 3.0 × 105 cells per well and incubated at 37°C for 12 h to allow cell attachment and growth. After the initial incubation, the cells were treated with compound 7 at its IC50 concentration dose value for 24 h. Following the treatment period, the cells were collected and fixed with 75% ethanol at 20°C overnight to arrest the cell cycle and preserve the cellular morphology. The fixed cells were then washed with phosphate-buffered saline (PBS) and centrifuged to remove the ethanol. Next, the cells were incubated with a solution containing ribonuclease (Rnase) at a concentration of 10 mg/mL and propidium iodide (PI) at a concentration of 5 mg/mL. Rnase is an enzyme that digests RNA, while PI is a fluorescent dye that stains DNA. The incubation with Rnase and PI allows for the detection of DNA content in the cells by flow cytometry analysis. After the incubation, the cells were subjected to flow cytometry analysis using a FACS Calibur cytometer with Cellquest software (BD Bioscience, USA). Flow cytometry analysis measures the fluorescence emitted by the cells stained with PI, which correlates with the DNA content. This analysis provides information about the cell cycle distribution of the treated cells, including the percentage of cells in different phases of the cell cycle (e.g., G0/G1, S, and G2/M phases), and allows for the assessment of any changes in the cell cycle profile induced by compound 7 treatment. To ensure the reliability of the results, the experimental conditions were repeated at least three times, and the cells were treated with compound 7 at its IC50 concentration dose value to ensure the cells were exposed to an effective concentration of the compound. This experimental method provided valuable information on the mechanism of action of compound 7 and its potential as an anticancer agent.

### 3.5 Annexin‐V‐FITC/PI assay for apoptosis assessment

The experimental method involved seeding HepG-2 cells at a density of 1.5 × 105 cells per well in a 6-well plate and incubating the cells for 12 h to allow for cell attachment and growth. Following this, the cells were treated with compound 7 at its IC50 concentration for 24 h to induce apoptosis. Apoptosis, a programmed cell death process, was detected by staining the cells with Annexin-V conjugated to fluorescein isothiocyanate (FITC) and Propidium Iodide (PI), and the stained cells were analyzed using a FACSCalibur cytometer and Cellquest software (BD Bioscience). Annexin-V is a protein that binds specifically to phosphatidylserine, a phospholipid that is externalized on the outer surface of the plasma membrane during early stages of apoptosis. FITC, a green-fluorescent dye conjugated to Annexin-V, is used to label the apoptotic cells. PI, a red-fluorescent DNA intercalating agent, is used to distinguish between apoptotic and necrotic cells based on their DNA content. The FACSCalibur cytometer is a flow cytometer used to analyze and quantify fluorescently labeled cells. The experimental method likely allowed for the quantification of apoptotic cells by analyzing the Annexin-V-FITC and PI staining patterns using flow cytometry. The results obtained from this method provided information on the induction of apoptosis by compound 7 in HepG-2 cells, further elucidating the mechanism of action of this compound as a potential anticancer agent. To ensure the reliability of the results, the experimental conditions were repeated at least three times, and the cells were exposed to therapeutically effective concentration of the compound, they were treated with compound 7 at a dose corresponding to its IC_50_ concentration. This ensured that the cells were subjected to an optimal concentration of the compound during the experiment. This experimental method yielded crucial information on the mechanism by which it exerts its anticancer effects.

### 3.6 Assessment of VEGFR-2 kinase activity

The VEGFR-2 inhibitory activity of compound 7 and Sorafenib was evaluated using human VEGFR-2 ELISA (enzyme-linked immunosorbent assay) kits, according to the manufacturer’s instructions. A 96-well plate was used for the assay, and each well was filled with a specific volume of the tested molecules and standard concentrations. The wells were coated with an immobilized antibody that specifically binds to VEGFR-2. After incubation, the wells were washed to remove unbound molecules, and biotinylated anti-human VEGFR-2 antibody was added to the wells. This biotinylated antibody specifically binds to VEGFR-2 that is captured by the immobilized antibody. Following another round of washing, a solution of conjugated streptavidin, which binds to the biotinylated antibody, was added to the wells. After washing to remove excess streptavidin, a solution of 3,3′,5,5′-tetramethylbenzidine (TMB) substrate was added to the wells and incubated for 30 min at 37°C. During this incubation, the bound conjugated streptavidin transformed the substrate into a colorful product. After terminating the reaction with an inhibitor solution, the optical density of the produced color was instantly determined at 450 nm with a spectrophotometer. The optical density values were used to quantify the inhibitory activity of compound 7 and Sorafenib against VEGFR-2, with higher inhibition resulting in lower optical density values. This assay provided a quantitative assessment of the inhibitory activity of compound 7 and Sorafenib against VEGFR-2 using an ELISA-based approach.

### 3.7 Assessment of cytochrome P450 (CYP2D6) activity

Human cells, obtained from the American Type Culture Collection (ATCC), were cultivated in RPMI medium (Invitrogen/Life Technologies). The medium was supplemented with 1% penicillin-streptomycin, 10 μg/ml of insulin (Sigma), and to support cell growth and 10% Fetal Bovine Serum (FBS) (Hyclone) viability. The cells were maintained at 37°C in a humidified atmosphere with 5% CO2. Cells were seeded into a 96-well plate at a density of 1.2–1.8 × 10,000 cells/well in a volume of 100 μL of complete growth medium. Compound 7 and SOR were added to the wells at various concentrations, ranging from low to high concentrations, to create a concentration-response curve. Each concentration was tested in triplicate. A control well with only complete growth medium was included as a negative control. The plate was incubated for 18–24 h at 37°C to allow the cells to adhere and grow in the presence of compound 7 and SOR. After the incubation period, the CYP inhibitory effect of compound 7 and SOR was evaluated using the Cytochrome P450 2D6 (CYP2D6) Inhibitor Screening Kit, following the manufacturer’s guidelines. The culture media was quickly withdrawn, and the cultured cells were rinsed with PBS. Then, 100 μL of the CYP2D6 enzyme substrate solution was added to each well, and the plate was incubated for a specified period of time (as recommended by the manufacturer) at 37°C. After the incubation, the fluorescence intensity of the converted product was measured using a FLx800™ Fluorescence Microplate Reader at the excitation wavelength of 390 nm and the emission wavelength of 488 nm. The fluorescence intensity data was used to generate concentration-response curves for compound 7 and SOR. The concentration that induced 50% maximal inhibition of CYP2D6 enzyme activity (IC50 value) was determined from the concentration-response curves using appropriate software or statistical methods. The IC50 values were calculated and used to quantify the inhibitory activity of compound 7 and SOR against CYP2D6 enzyme. The data were analyzed for statistical significance and presented as mean ± standard deviation (SD) from triplicate measurements.

### 3.8 Molecular docking study

Extensive *in silico* molecular docking investigations was conducted using MOE software to assess the affinity for attachment of this chemical class to the function sites of VEGFR2 and CYP2D6 proteins. Molecular docking enables the assessment of the binding affinity between small molecules and the binding site of the targeted protein, providing valuable insights into the mode of action of pharmacological compounds. To evaluate the binding mode of the designed N-(substituted-phenyl)-8-methoxycoumarin-3-carboxamide analogues, compound **7** was particularly selected to investigate its binding affinity towards the functional pocket of VEGFR-2 and CYP2D6 proteins. The 2D structure of compound **7** was acquired using Chem.Draw software, allowing for additional computer investigation of the chemical compound. The Protein Data Bank (PDB) provides a wealth of crystallographic structures for both VEGFR2 and CYP2D6 proteins, facilitating our study. Specifically, we selected the crystal structure of VEGFR2 protein co-crystallized with Sorafenib (PDB code: *4asd*) and the crystal structure of CYP2D6 protein co-crystallized with BACE1 inhibitor 6 (PDB code: *4xrz*) (McTigue et al., 2012; Brodney et al., 2015). These structures offer detailed insights into the three-dimensional arrangement of the proteins, enabling a comprehensive analysis of their structural features and potential binding sites. To prepare the 3D structures of VEGFR2 and CYP2D6 proteins for docking simulations, several steps were taken. First, the structures were protonated to account for the ionization states of amino acid residues at a specific pH. Next, partial charges were assigned to the atoms, and any additional chains and water molecules were removed from the structures to focus solely on the protein of interest. The MMFF94X force field was used to represent the distribution of charges within proteins, and energy minimization was carried out to optimize their conformations. To ensure the reliability and accuracy of the docking process, adjustments were made to the docking protocol. These adjustments involved employing Triangle Matcher placement and the London dG scoring function. The customized protocol aimed to improve the accuracy of predicting ligand binding. To confirm the effectiveness of the modified protocol, the binding affinity and interaction mode of the original co-crystallized ligand were carefully analyzed and compared to the reported data. This comparison served as a benchmark to assess the reliability of the docking results. Following the docking simulations, a thorough evaluation was performed on the resulting data. The binding modes exhibiting significantly strong binding affinity were specifically identified and chosen for subsequent analysis, taking into consideration their potential enlightenment to the desired targets. These selected binding modes were then used to estimate the corresponding docking scores and binding energies, providing quantitative measurements of the ligand-receptor interactions. By employing this rigorous docking approach, incorporating Triangle Matcher placement and the London dG scoring function, the validity and accuracy of the docking predictions were enhanced. The evaluation of the original ligand’s binding interactions and the subsequent selection of high-affinity binding modes allowed for a comprehensive analysis of the ligand’s potential binding affinity and energetics. In our analysis, we generated a total of 50 conformations crossponding to each protein, which were further assessed to examine the interactions between the ligand (compound 7) and amino acid residues. Additionally, the binding energy of each pose was assessed to quantify the strength of the ligand-protein interactions and provide insights into the stability and potential affinity of the compound within the binding site.

### 3.9 *In silico* ADME and toxicity prediction

In order to comprehensively assess the pharmacokinetic properties, drug-likeness, and potential toxicity of compound 7, an *in silico* computational evaluation and prediction were conducted. This evaluation involved comparing compound 7 with the reference drugs sorafenib and staurosporine. To perform these assessments, several computational tools were employed, including SwissADME, Protox II, OSIRIS Property Explorer, and pkCSM (Alzahrani et al., 2022; Abdelgalil et al., 2023; Hassan et al., 2023; Ragab et al., 2023). These tools have been widely used and established in the field for predicting various pharmacological and physicochemical properties of small molecules. SwissADME was employed to forecast essential pharmacokinetic variables, encompassing absorption, distribution, metabolism, excretion, and toxicity (ADMET). Protox II was employed to predict the potential toxicity of compound 7 by analyzing its interaction with various protein targets. OSIRIS Property Explorer was utilized to evaluate drug-likeness and predict the likelihood of compound 7 possessing specific toxicological, physicochemical, and environmental properties. Lastly, pkCSM was employed to assess the compound’s potential to bind to different proteins and predict its pharmacokinetic properties. By utilizing these computational tools, a comprehensive evaluation of compound 7 was conducted, considering its pharmacokinetic properties, drug-likeness, and potential toxicity. The comparison with the reference drugs sorafenib and staurosporine allowed for a relative assessment of compound 7’s profile. These computational predictions contribute to the initial characterization of compound 7 and provide valuable insights into its potential as a therapeutic agent (Alzahrani et al., 2022; Abdelgalil et al., 2023; Hassan et al., 2023; Ragab et al., 2023).

## 4 Conclusion

Liver cancer continues to be a prominent issue in global health, representing the sixth most frequently diagnosed cancer and the fourth highest contributor to cancer-related fatalities on a global scale. The current study focused on investigating the antitumor potency of a novel class of compounds, specifically N-(substituted-phenyl)-8-methoxycoumarin-3-carboxamides, against liver cancer. The compounds were designed, synthesized, and characterized to assess their potential as effective anticancer agents. The assessment of antitumor activity revealed that the synthesized class of compounds exhibited significant cytotoxicity against Hep-G2 cells, a liver cancer cell line, surpassing the efficacy of the drug staurosporine while exhibiting minimal impact on normal cells. Compound 7 exhibited the highest cytotoxic activity against Hep-G2 cells among the synthesized compounds, displaying an IC_50_ value of 0.75 μM, which outperformed staurosporine’s cytotoxicity (IC_50_ = 8.37 μM). Further investigation into the mechanism of action of compound **7** unveiled its ability to interfere with DNA replication, induce DNA damage, and consequently lead to cell cycle arrest. Specifically, compound **7** significantly reduced the percentage of cells in the G1 and G2/M phases while increasing the percentage of cells in the S phase. Additionally, flow cytometric analysis indicated that compound **7** triggered programmed cell death through the induction of necrosis and apoptosis in HepG-2 cells. Moreover, compound **7** exhibited a dual-inhibitory activity towards vascular endothelial growth factor receptor-2 (VEGFR-2) and cytochrome P450 enzymes, surpassing the activity of the drug sorafenib. Computational studies provided further insights by revealing a substantial binding affinity of compound **7** towards the binding cavity of VEGFR-2 and CYP450 enzymes. The study findings strongly indicate that the presented class of compounds, notably compound **7**, presents an exciting opportunity as a scaffold for developing exceptionally efficacious agents against liver cancer. These compounds exhibit immense potential for advancing novel therapeutics that can effectively combat liver cancer. The synthesized compounds exhibited remarkable cytotoxic effects on liver cancer cells by disrupting DNA replication, triggering programmed cell death, and demonstrating dual-inhibitory efficacy against VEGFR-2 and CYP450 enzymes. These findings emphasize the potential of these compounds as innovative therapeutic candidates for the treatment of liver cancer. Future research can focus on further optimizing the structure of compound **7** and conducting *in vivo* studies to validate its efficacy and safety profile as a potential liver cancer treatment option.

## Data Availability

The original contributions presented in the study are included in the article/Supplementary Material, further inquiries can be directed to the corresponding authors.
