# Supplementary material for: Unveiling the antitumor potential of novel N-(substituted-phenyl)-8-methoxycoumarin-3-carboxamides as dual inhibitors of VEGFR2 kinase and cytochrome P450 for targeted treatment of hepatocellular carcinoma
Source: Front Chem. 2023 Aug 4;11:1231030. doi: 10.3389/fchem.2023.1231030 (PMC10436493; doi:10.3389/fchem.2023.1231030)
Supplement: Supplementary file 1 [file DataSheet1.pdf]

Supplementary Materials

Unveiling the Antitumor Potential of Novel *N*-(Substituted-Phenyl)-8-Methoxycoumarin-3-Carboxamides as Dual Inhibitors of VEGFR2 Kinase and Cytochrome P450 for Targeted Treatment of Hepatocellular Carcinoma

Eman M. Radwan [1], Eman Abo-Elabass [2], Atef E. Abd El-Baky [3], Hussah Abdullah Alshwyeh [4,5], Riyad A. Almaimani [6], Ghassan Almaimani [7], Ibrahim Abdel Aziz Ibrahim [8], Abdulaziz Albogami [9], Mariusz Jaremko [10,*], Samar Z. Alshawwa [11], Essa M. Saied [12,13,*]

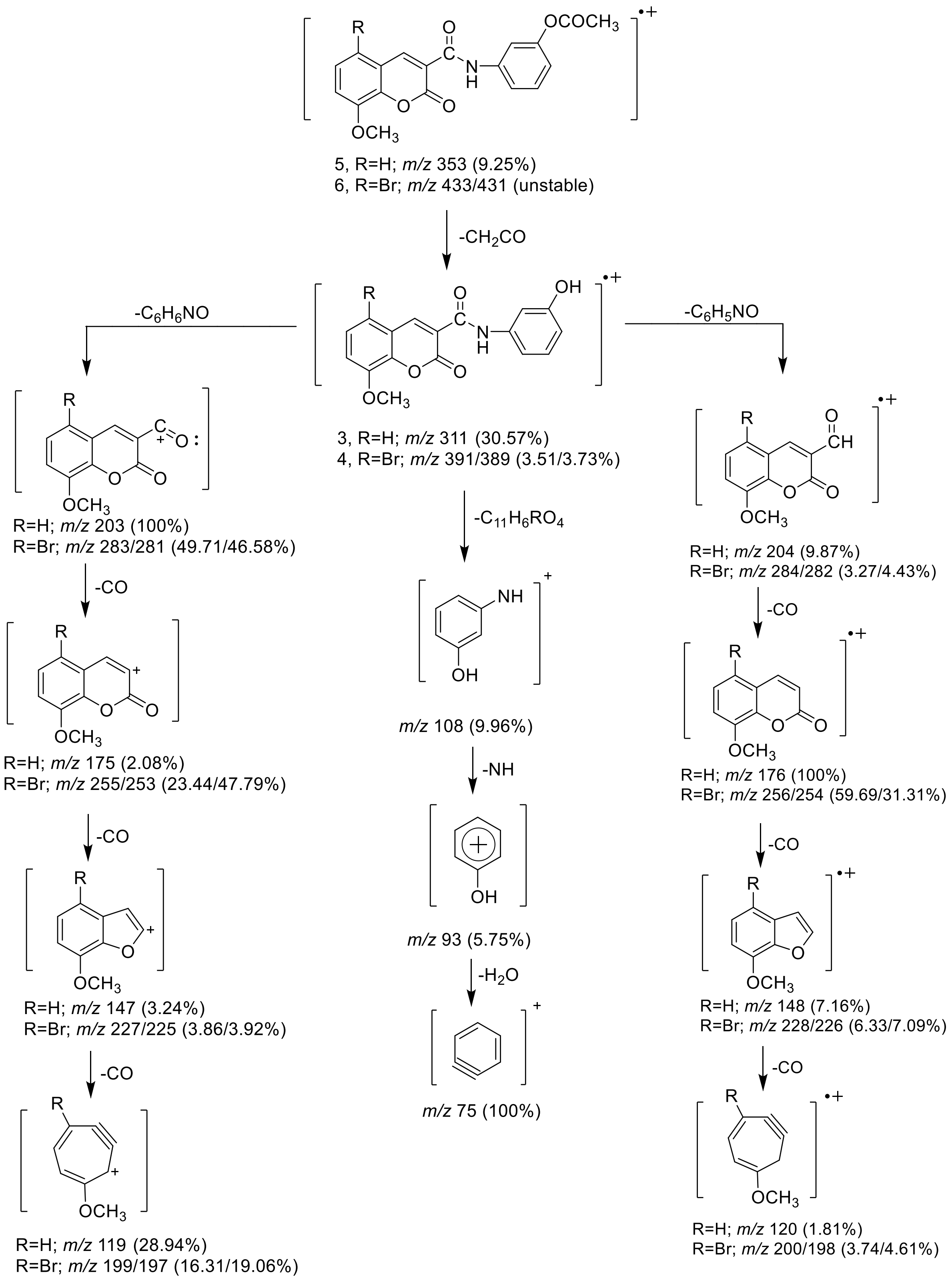

Scheme S1. The fragmentation pathways of compounds **3-6**.

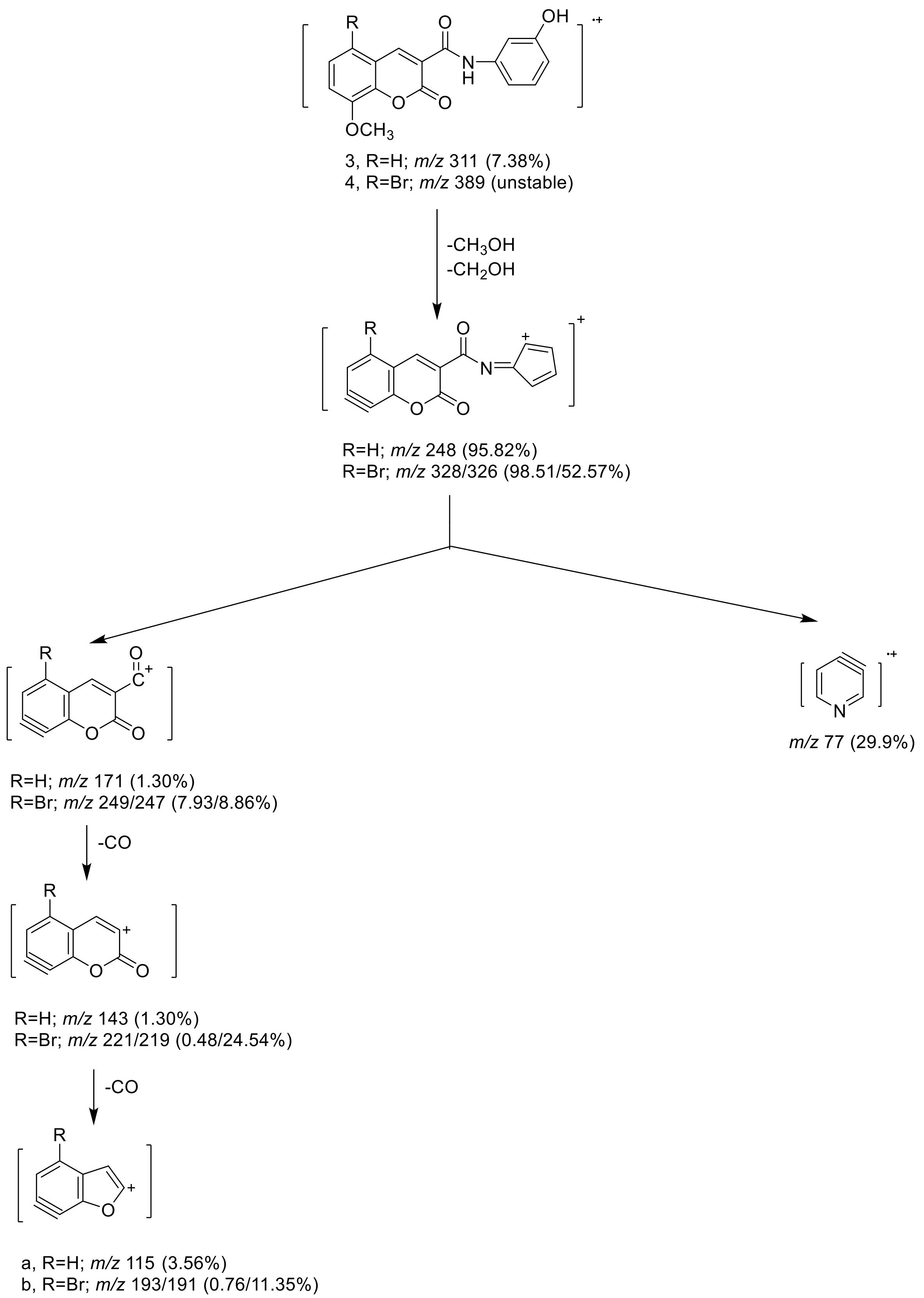

Scheme S2. The fragmentation pathways of compounds **3-4**.

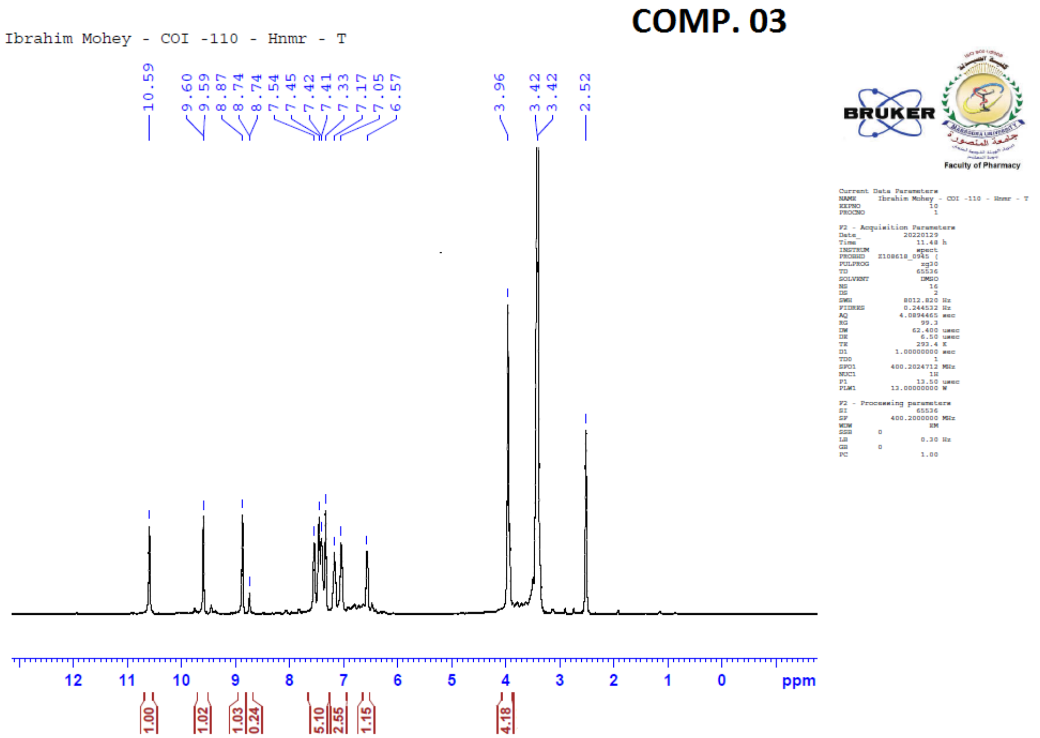

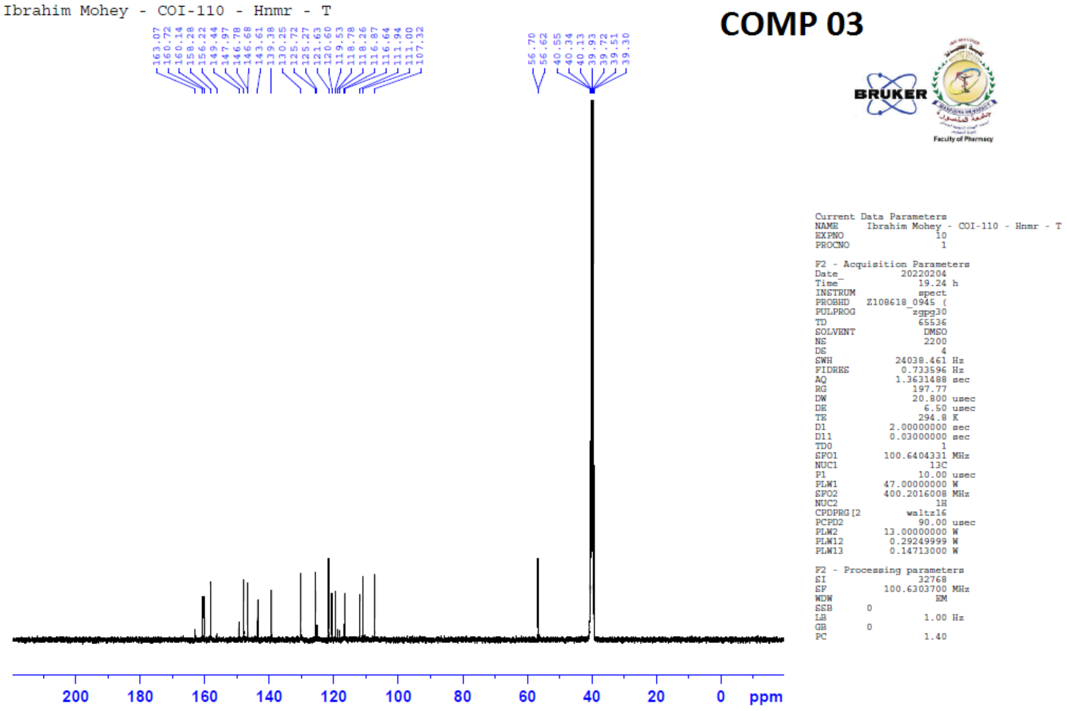

Figure S1. ^{1}H NMR and ^{13}C NMR spectra of compound **3**.

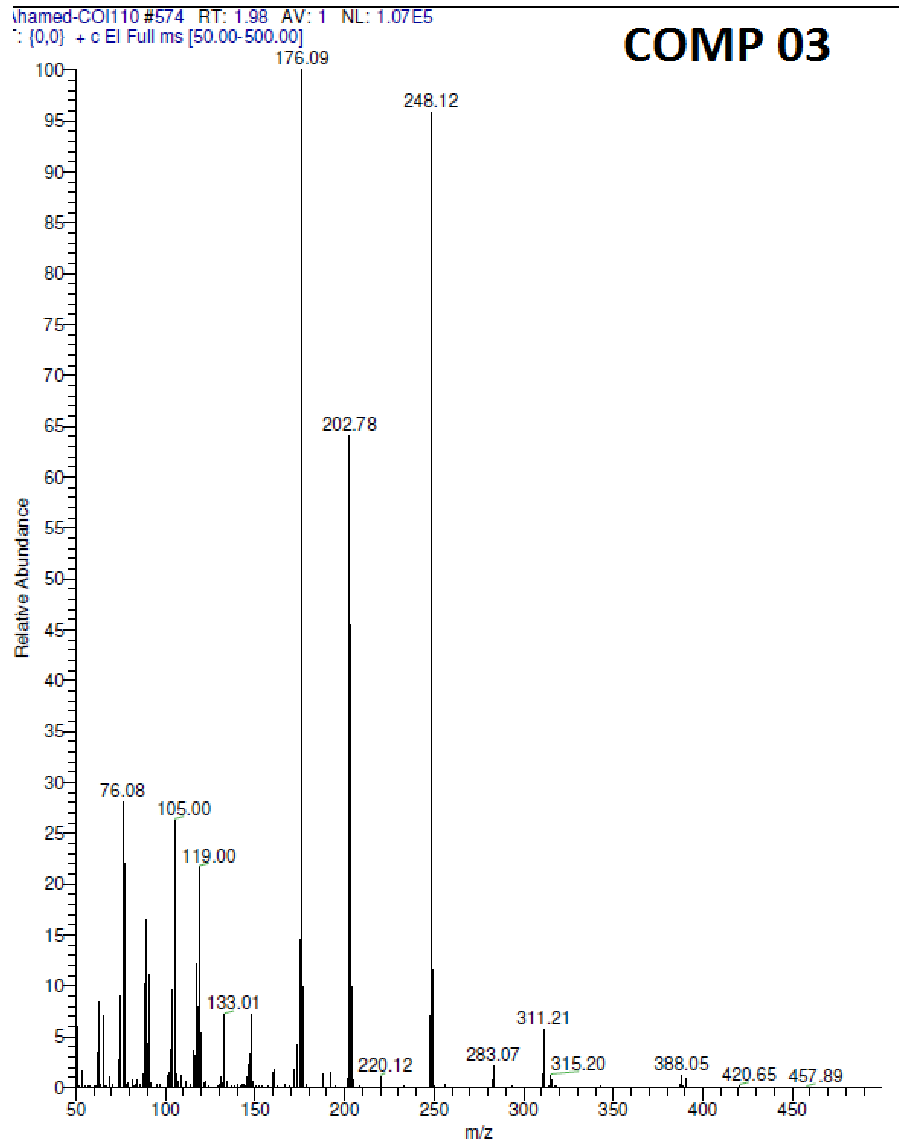

Figure S1b. Mass spectrum of compound **3.**

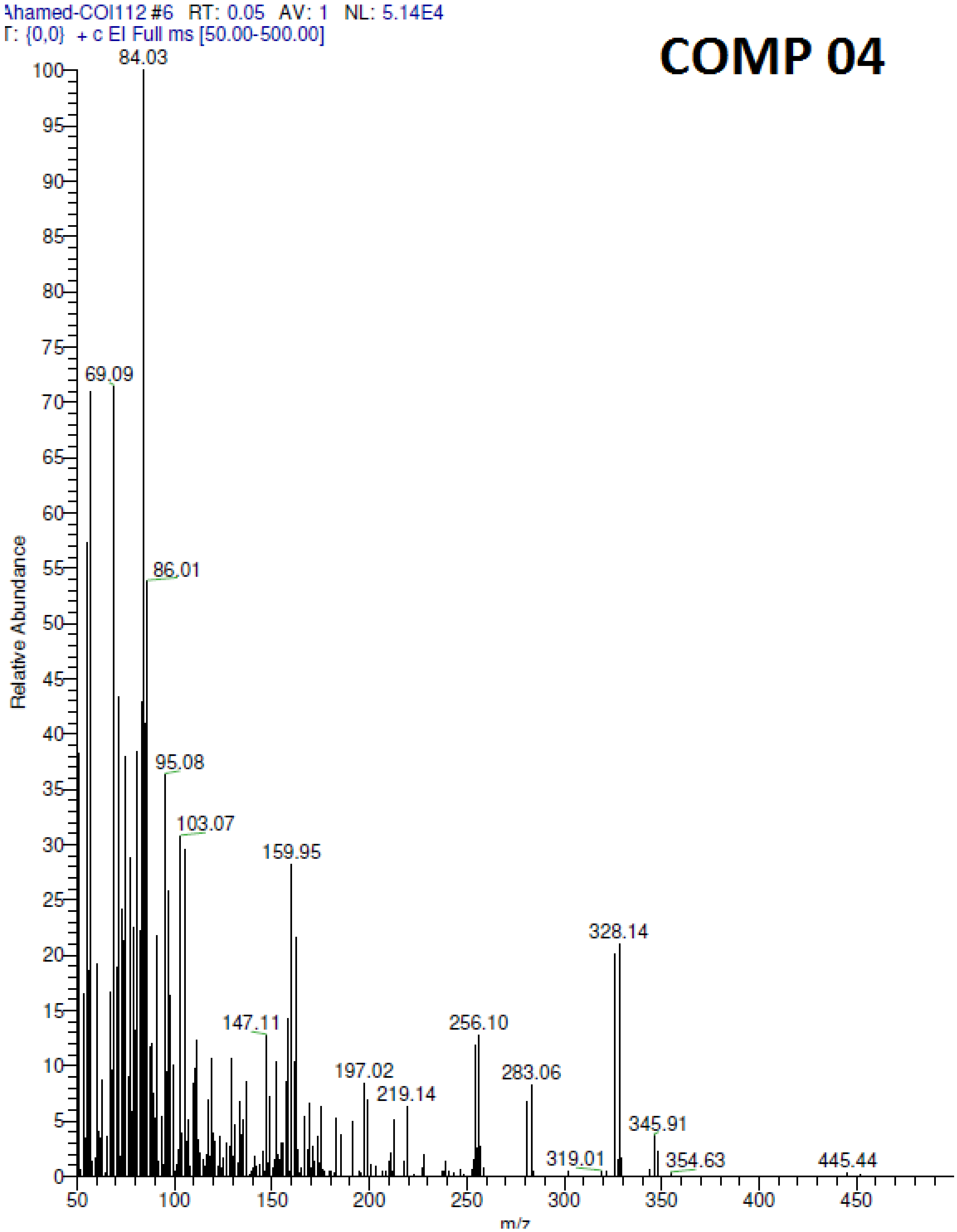

Figure S2. Mass spectrum of compound **4.**

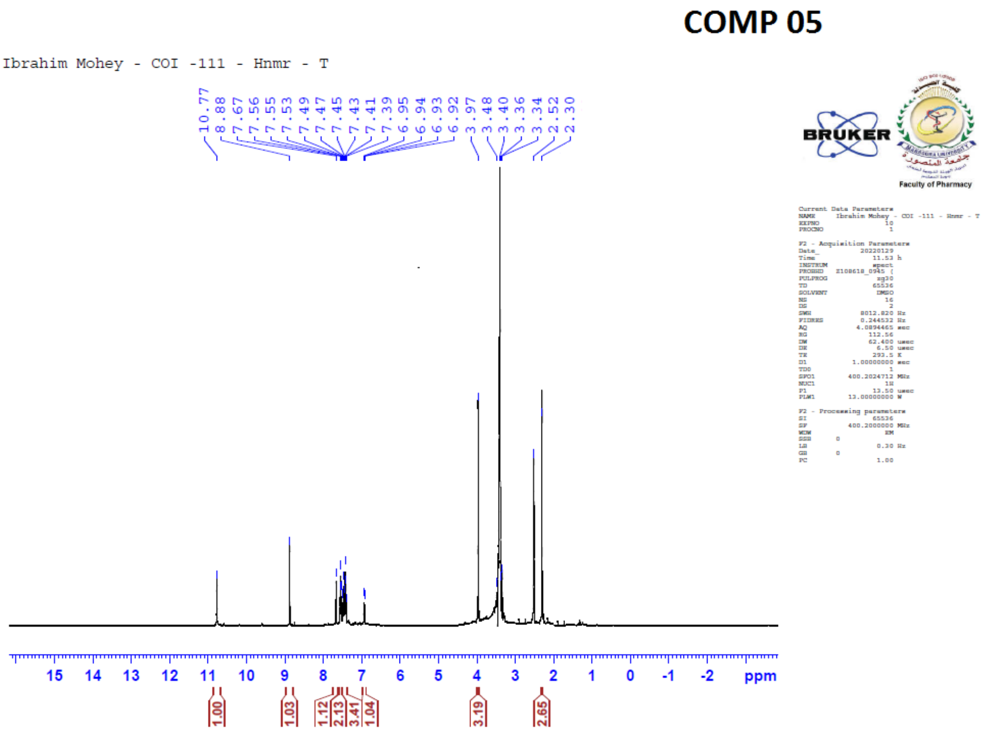

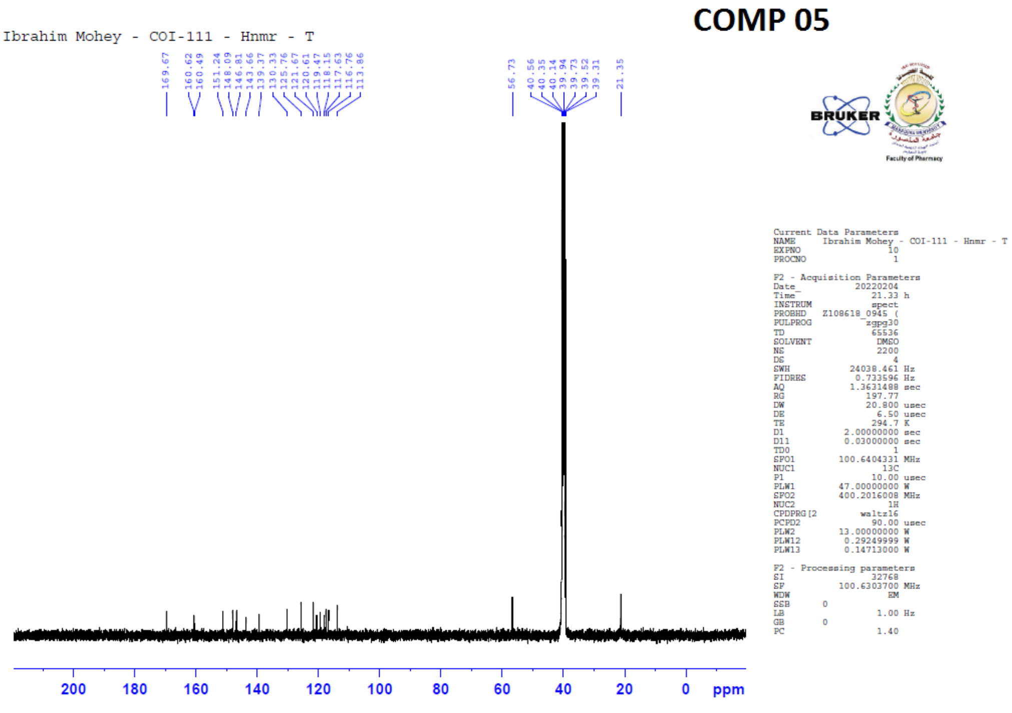

Figure S3. ^{1}H NMR and ^{13}C NMR spectra of compound **5.**

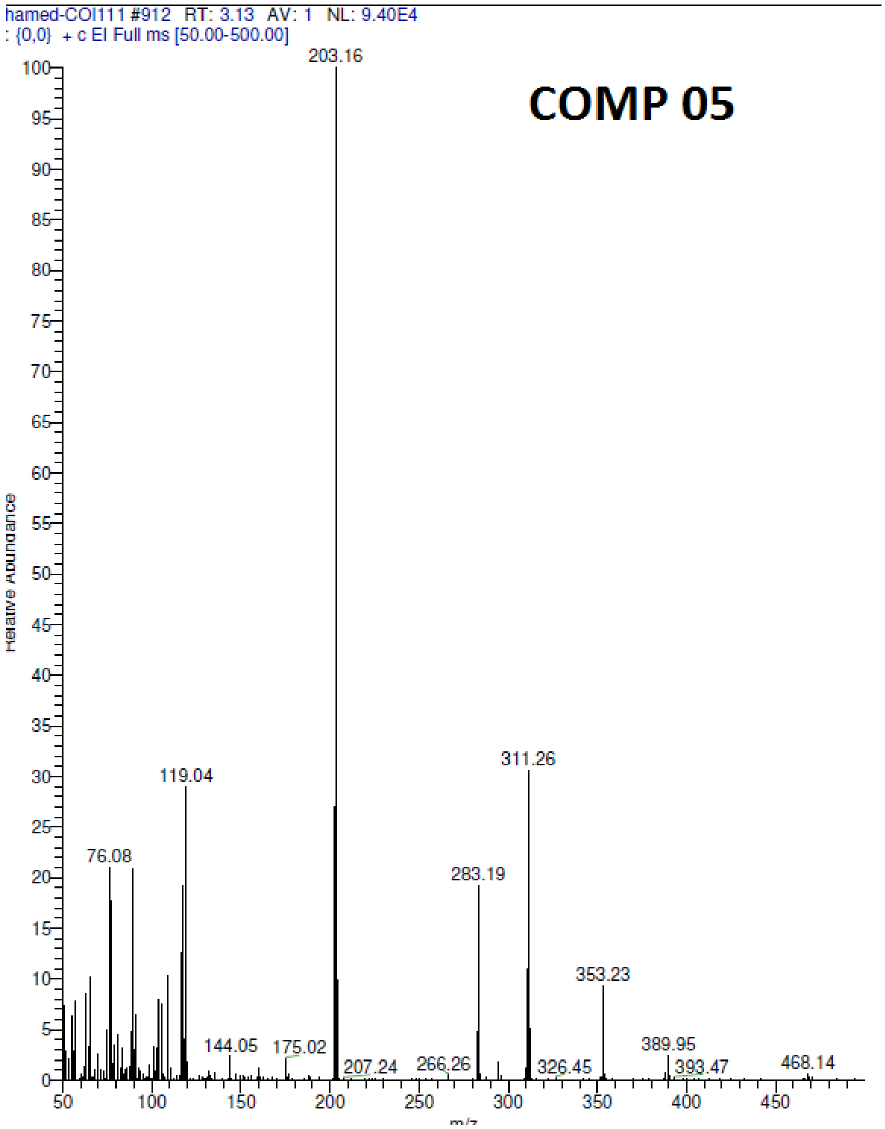

Figure S3b. Mass spectrum of compound **5.**

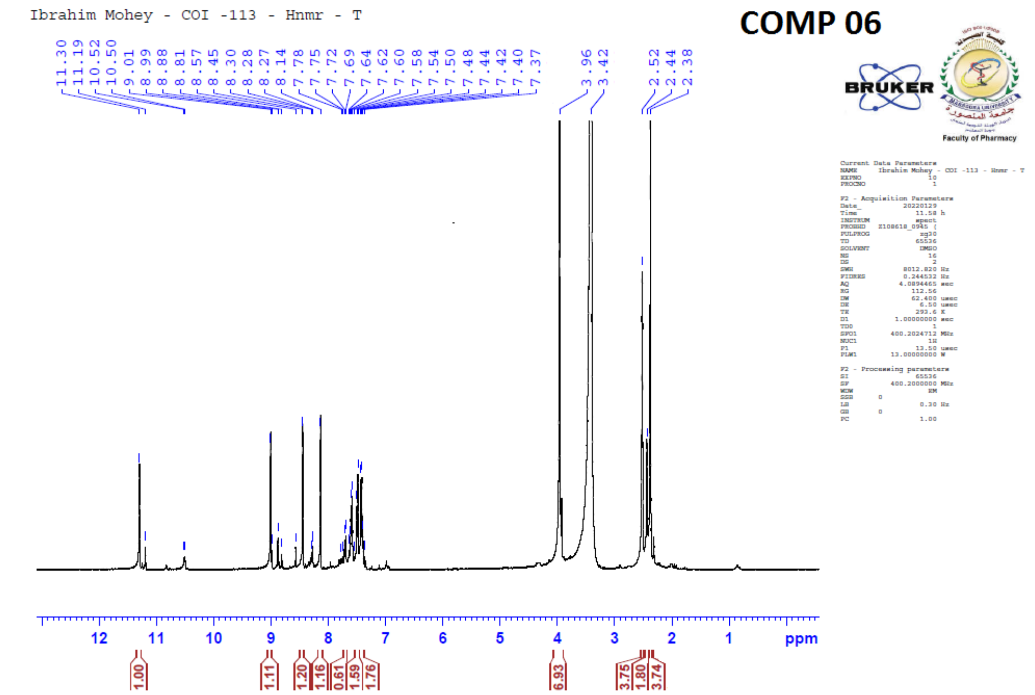

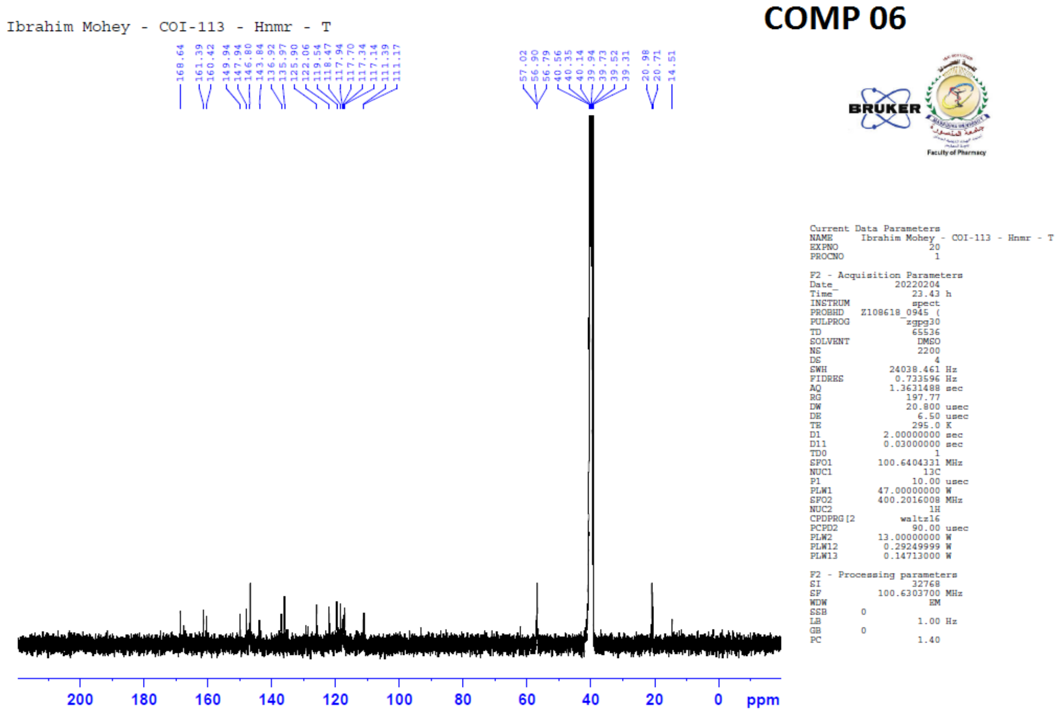

Figure S4. ^{1}H NMR and ^{13}C NMR spectra of compound **6.**

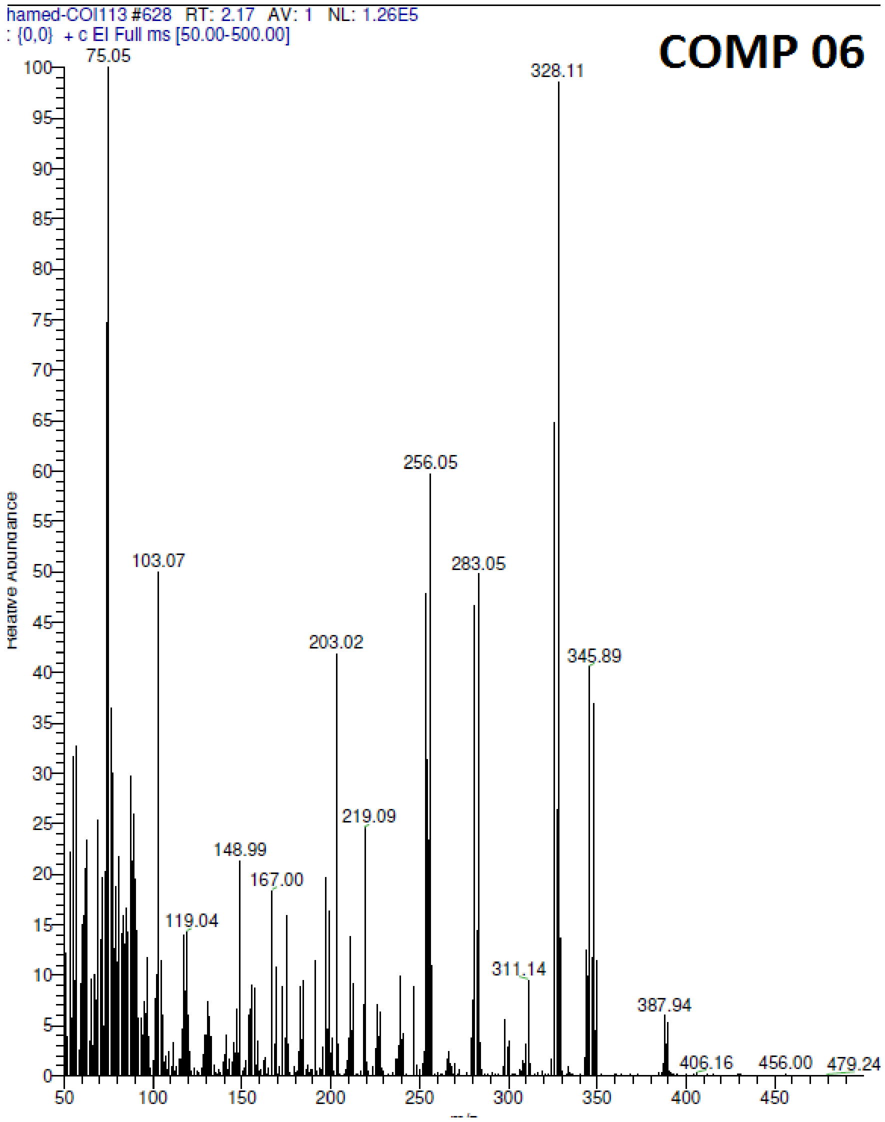

Figure S4b. Mass spectrum of compound **6.**

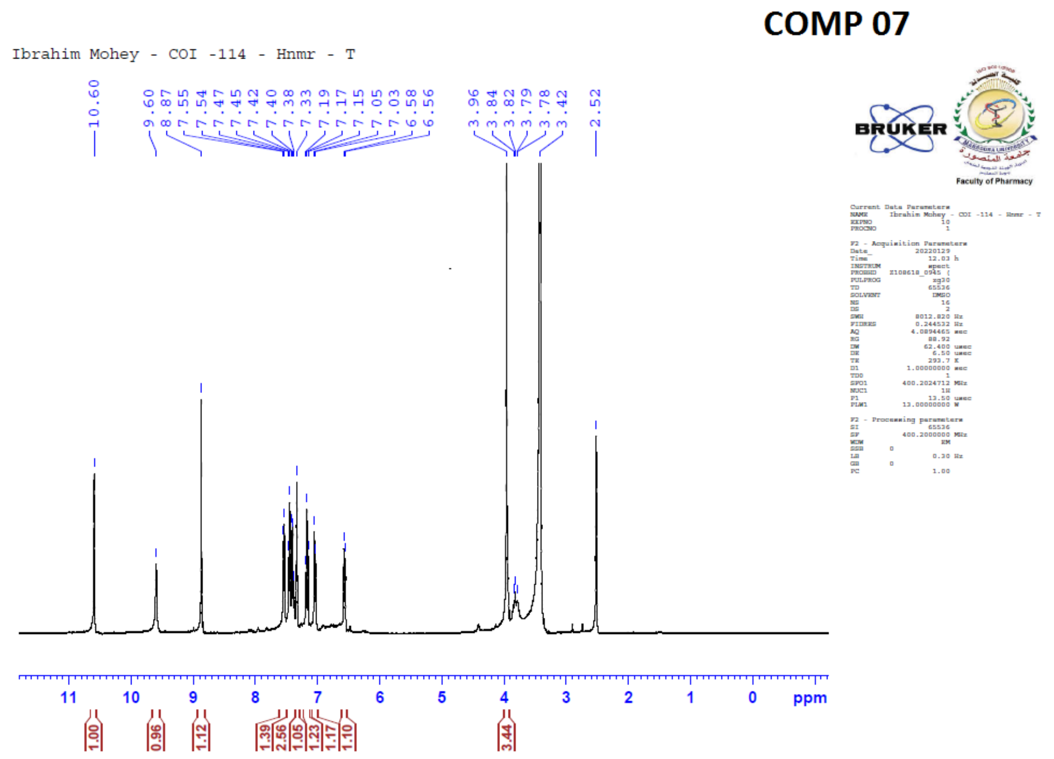

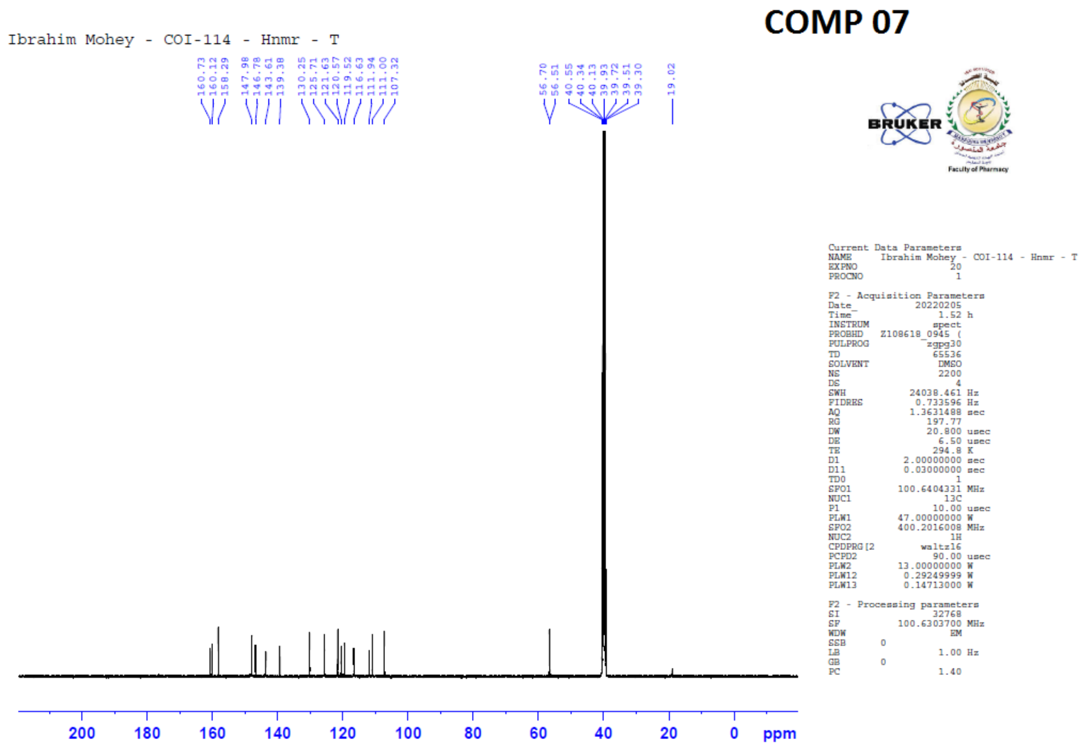

Figure S5. ^{1}H NMR and ^{13}C NMR spectra of compound **7.**

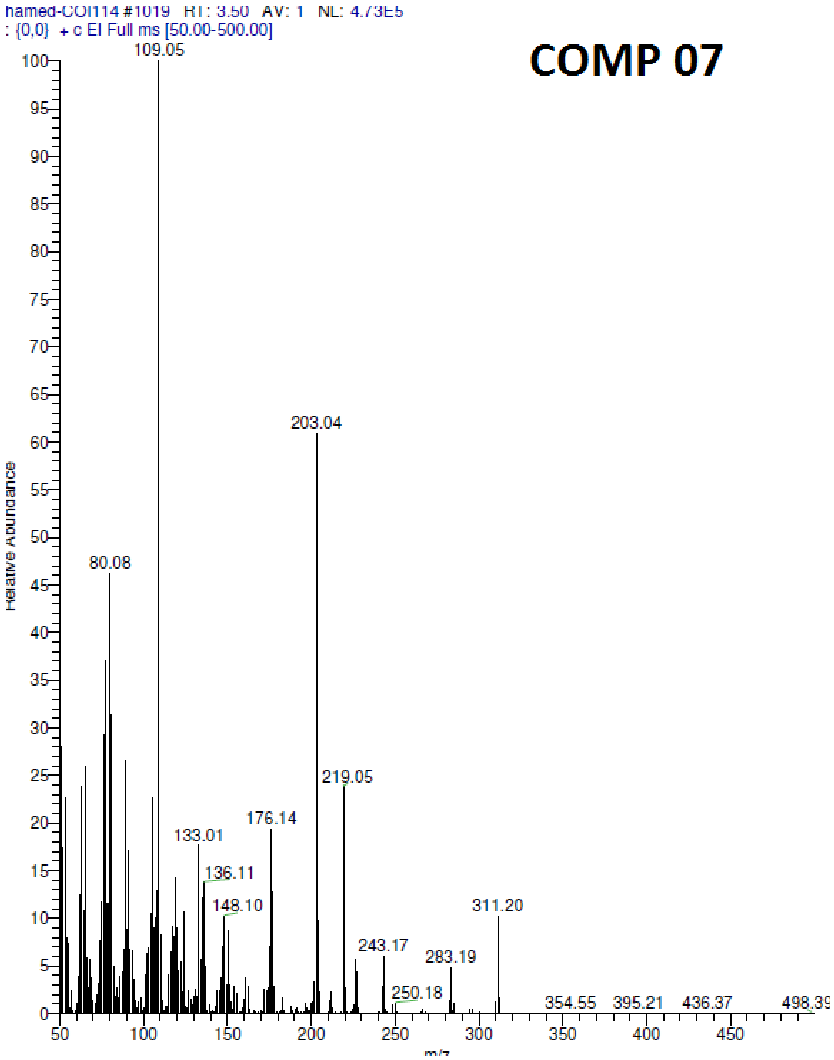

Figure S5. Mass spectrum of compound **7.**

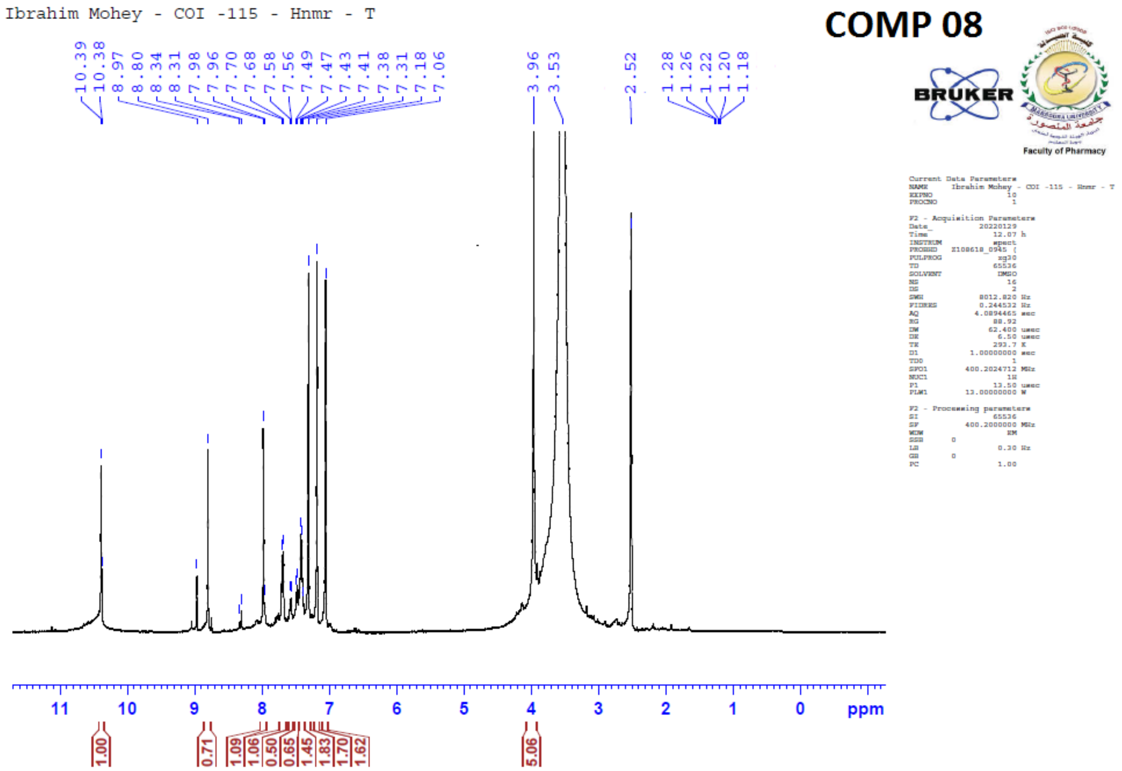

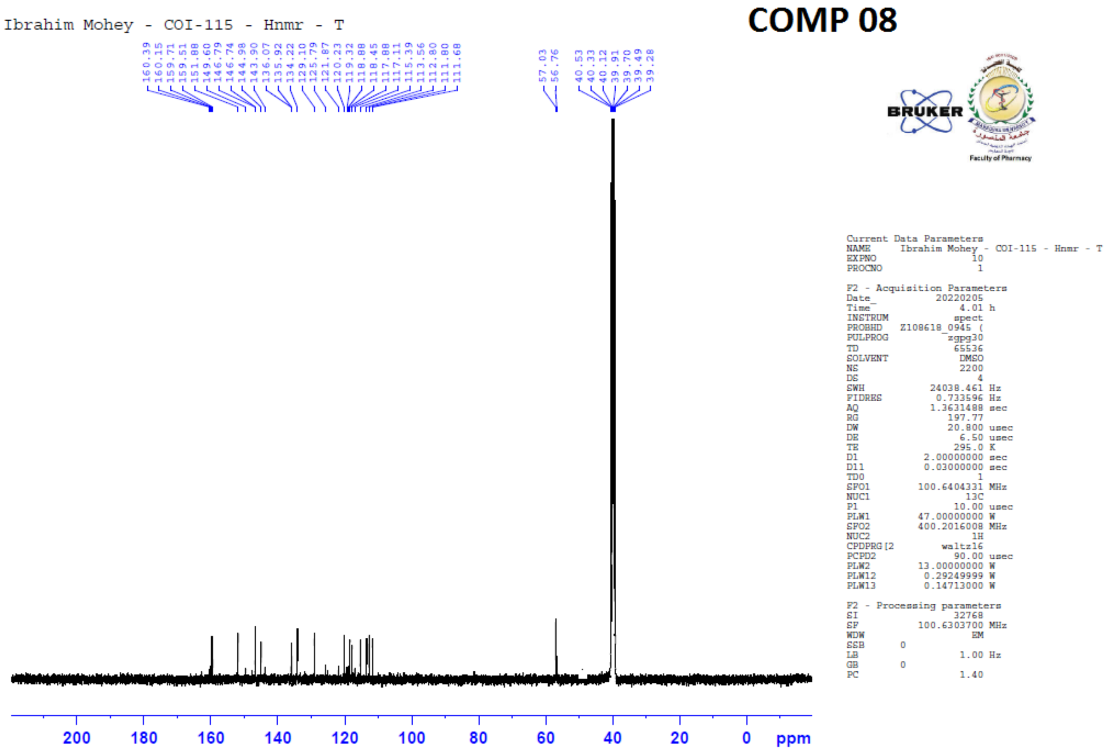

Figure S6. ^{1}H NMR and ^{13}C NMR spectra of compound **8.**

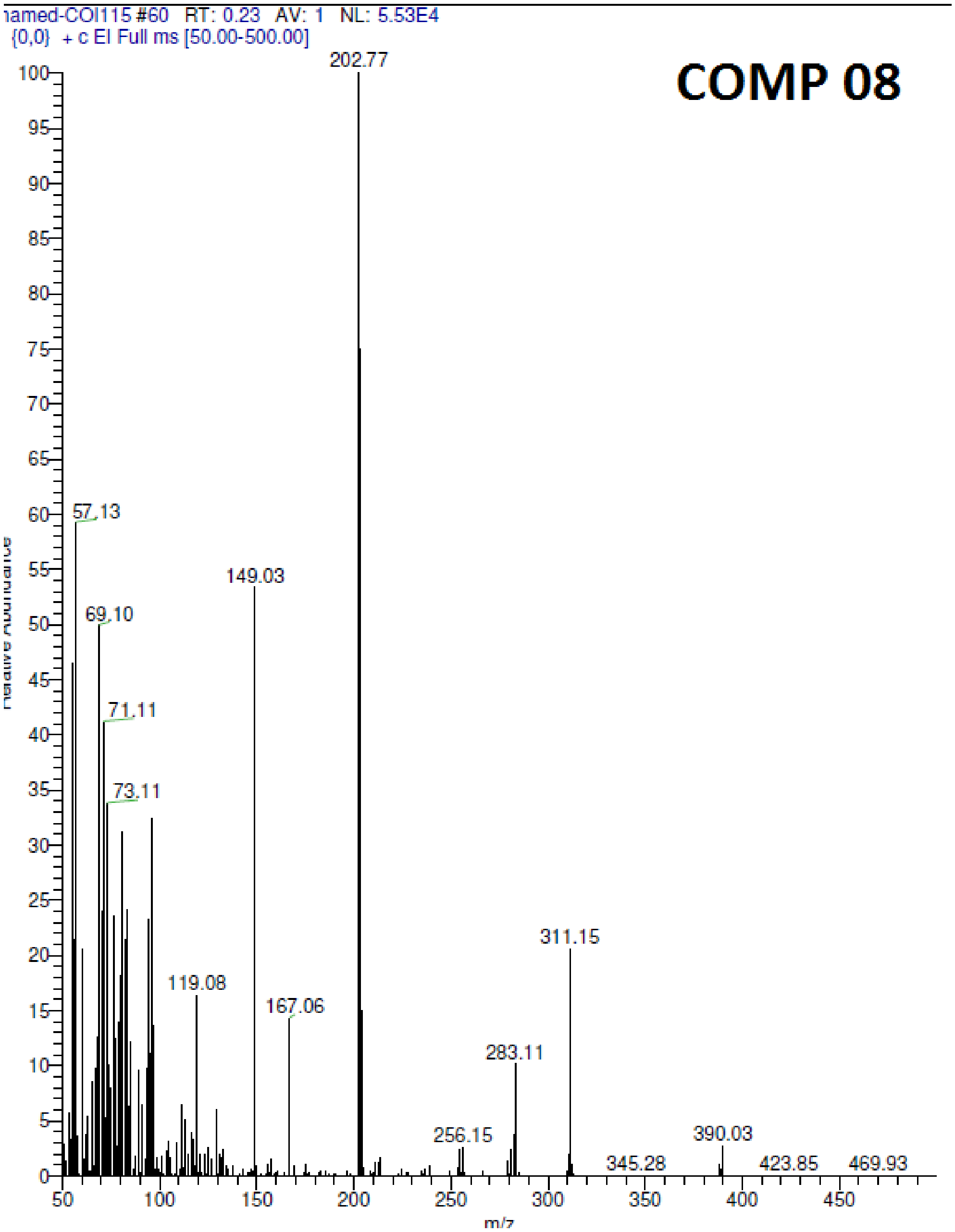

Figure S6b. Mass spectrum of compound **8.**
